# Identification of Genes Essential for Fluorination and Sulfamylation within the Nucleocidin Gene Clusters of *Streptomyces calvus* and *Streptomyces virens*

**DOI:** 10.1002/cbic.202200684

**Published:** 2023-01-26

**Authors:** Marta Wojnowska, Xuan Feng, Yawen Chen, Hai Deng, David O'Hagan

**Affiliations:** ^1^ School of Chemistry University of St Andrews St Andrews Fife KY16 9ST UK; ^2^ Department of Chemistry University of Aberdeen Aberdeen AB24 3UE UK

**Keywords:** nucleocidin, fluorometabolite biosynthesis, Streptomyces, gene knockouts, natural products

## Abstract

The gene cluster in *Streptomyces calvus* associated with the biosynthesis of the fluoro‐ and sulfamyl‐metabolite nucleocidin was interrogated by systematic gene knockouts. Out of the 26 gene deletions, most did not affect fluorometabolite production, nine abolished sulfamylation but not fluorination, and three precluded fluorination, but had no effect on sulfamylation. In addition to *nucI, nucG*, *nucJ*, *nucK*, *nucL*, *nucN*, *nucO*, *nucQ* and *nucP*, we identified two genes (*nucW*_,_ *nucA)*, belonging to a phosphoadenosine phosphosulfate (PAPS) gene cluster, as required for sulfamyl assembly. Three genes (*orf(−3)*, *orf2* and *orf3*) were found to be essential for fluorination, although the activities of their protein products are unknown. These genes as well as *nucK*, *nucN*, *nucO* and *nucPNP*, whose knockouts produced results differing from those described in a recent report, were also deleted in *Streptomyces virens* – with confirmatory outcomes. This genetic profile should inform biochemistry aimed at uncovering the enzymology behind nucleocidin biosynthesis.

## Graphical abstract

**A knockout screen** along the biosynthetic gene cluster associated with nucleocidin **1** biosynthesis led to different outcomes from a study reported recently. For example, at least 11 genes are involved in installing the sulfamyl moiety on the ribosyl ring of nucelocidin and at least 4 gene are required for fluorometabolite production.

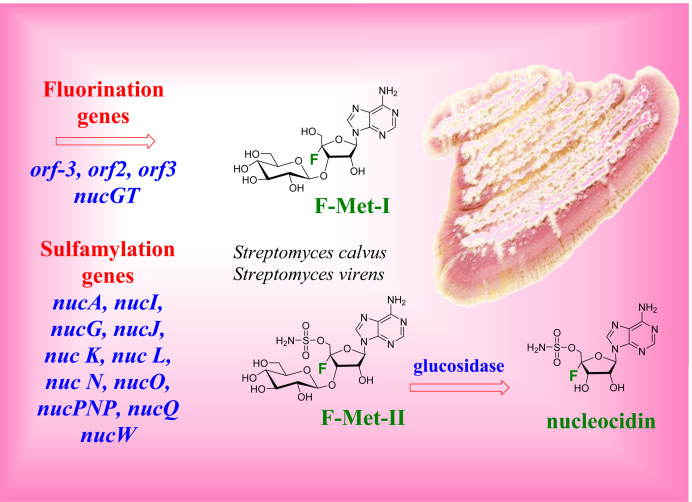

## Introduction

Nucleocidin **1** is a naturally occurring modified adenosine nucleoside.^[1]^ It was originally isolated from the actinomycete bacterium *Streptomyces calvus*, due to its anti‐trypanosomal activity, although it proved too toxic to develop as a therapeutic.^[2]^ Today it attracts particular attention as it belongs to the unique group of natural products that contain a fluorine atom. Only a handful of such fluorometabolites are known, of which the most notable is fluoroacetate, a metabolite found in a wide range of tropical and sub‐tropical plants and some bacteria, including *Streptomyces* sp.^[3]^ The enzymology behind C−F bond formation in fluoroacetate biosynthesis in bacteria is reasonably well understood, involving the action of adenosyl‐fluoride synthase (EC. 2.5.1.63‐fluorinase), an enzyme which combines *S*‐adenosyl‐l‐methionine and a fluoride ion to form 5’‐fluorodeoxyadenosine and l‐methionine.^[4]^ 5’‐Fluorodeoxyadenosine is then biochemically processed to fluoroacetate and a range of related metabolites.^[5]^ However the mechanism behind the biosynthesis of nucleocidin **1**, and particularly the introduction of the fluorine atom at the 4’‐position of the ribose ring, is unknown. An understanding of this biosynthetic pathway merits attention as there are prospects of developing a biotechnology towards C−F bond formation from fluoride,^[6]^ as well as accessing the unusual 4’‐fluororibose structural motif by this approach. Ribonucleosides with a fluorine at the 4’‐position present a synthetic challenge as the fluorine is located at a tertiary centre and the anomeric nature of the motif renders it rather unstable and sensitive to conditions.^[7]^ As a consequence this class of fluoro‐nucleosides has received rather limited attention, although recently there has been some progress in exploring 4’‐fluororibose compounds as antivirals.^[8]^ Meanwhile, fluoro‐nucleosides more generally – particularly those with fluorine at the 2’‐ and 3’‐positions of the ribose moiety – have been extensively investigated, and there are many examples of extremely successful clinical bioactives in the context of oncology and antiviral therapies.^[9]^ Notwithstanding the potential for a fluorination biotechnology, nucleocidin also contains a sulfamyl group – another common substituent in medicinal chemistry, yet an exceedingly rare motif in natural products.^[10]^ Therefore an understanding of the biosynthesis of this unique natural product offers the downstream potential of bio‐transformations to introduce these two attractive substituents into building blocks for drug discovery.

**Figure 1:**
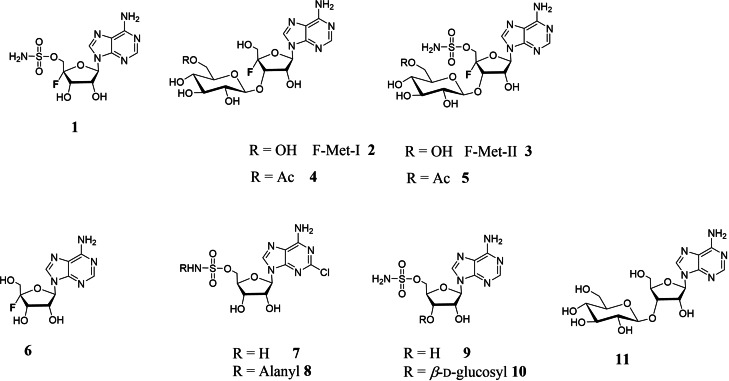
Structures of the known 4’‐fluoroadenosine metabolites and sulfamyl containing natural products.

Nucleocidin **1** is structurally related to the sulfamylated antibiotic ascamycin **8** (Figure 1).[^11^, ^12^] Interestingly, **8** and its co‐metabolite, dealanylascamycin **7**, also contain a halogen – a chlorine atom located at C‐2 of the adenine ring. Partial genome sequencing has allowed identification of the biosynthetic gene cluster (BGC) for **7** and **8**, which was inferred from the co‐localisation of several genes encoding putative sulfate processing enzymes.^[11]^ Knockouts of two such genes (*acmG* and *acmK*) in the genome of the **7** and **8** producer, *Streptomyces* sp. JCM9888, disabled antibiotic production, strongly suggesting that the genes in that cluster encode sulfamylation enzymes. Homologues of some of these genes were then discovered in the **1** producer, *S. calvus*, indicating the location of **1** BGC,^[13]^ however for several decades nucleocidin **1** could not be obtained in fermentations of the publicly available strains of *S. calvus*. It emerged that this lack of production was associated with a bald phenotype resulting from a mutation in the *bldA* gene, which disabled translation of the rare TTA codon found in several genes of the **1** BGC.^[14]^ Correction of that mutation re‐established production of **1** and facilitated the functional verification of the BGC. Its role was further validated by the discovery that other *Streptomyces* strains containing the cluster – *S. asterosporus* DSM41452,^[15]^ *S. aureorectus* DSM41692^[16]^ and *S. virens* DSM41465^[16]^ – also produce nucleocidin **1** in culture. Nucleocidin **1** appears to be the primary fluorinated bioactive produced by these organisms; however, several other 4’‐fluoroadenosine analogues related to nucleocidin have been identified as co‐metabolites in the cultures of the producing organisms. Most significant are F‐Met‐I **2** and F‐Met‐II **3** which have a β‐glucosyl moiety attached to the 3’‐OH of the ribosyl of adenosine.^[17]^ Notably, **2** lacks the sulfamyl group whereas **3** carries both sulfamyl and fluorine substituents. These two fluorometabolites appear simultaneously in batch fermentations of the producing organisms and their gradual disappearance is concomitant with the emergence and accumulation of **1**, suggesting that they may constitute biosynthetic precursors of **1**. β‐Glucosylation is a common strategy used by microorganisms which export bioactive natural products out of the cell,^[18]^ and this might be the reason why **2** and **3** are glucosylated; however, the biosynthetic relationships between these metabolites and **1** are not clear at present. Most recently Pasternak *et al*.^[19]^ have reported the isolation of **4** and **5** – acetylated derivatives of **2** and **3** in *S. virens* – and provided biochemical evidence for the enzymatic activity responsible for this O‐acetyl modification converting **2** and **3** to **4** and **5**, respectively. At this stage it is not known whether this modification is relevant to nucleocidin activity, given the very low abundance of the acetylated metabolites. We have also identified de‐fluoro analogues of the sulfamylated metabolites, **9** and **10**, as well as the defluorinated glucosyl adenosine **11** from *S. calvus*.^[20]^ The presence of **9**–**11** at approximately half the level of their corresponding 4’‐fluorometabolites suggests that the glucosylation and sulfamylation processes operate independently of fluorination.

We have conducted an exhaustive gene knockout screen along the nucleocidin **1** BGC in *S. calvus* to investigate the relationship between individual genes and metabolite production and are prompted to report our observations to date following a recent disclosure by Pasternak *et al*.,^[19]^ who have described their findings from a more limited gene disruption screen in *S. virens*. Their study identified two sets of genes, with the first set (*nucJ, nucG* and *nucI*) implicated in the assembly of the sulfamyl moiety and the second set (*nucN, nucK* and *nucO*) identified as essential for the incorporation of fluorine. Another gene, *nucPNP*, was also previously implied as essential for fluorometabolite production.^[14]^ Our data support these observations with respect to sulfamylation; however, there are substantial differences with respect to the genes/enzymes involved in the fluorination process. We report on the outcome of 26 individual gene knockouts in the **1** BGC of *S. calvus*, of which seven were additionally verified by analogous knockouts in *S. virens*. Surprisingly, as many as nine enzymes encoded in this BGC are necessary for the sulfamylation process. Furthermore, we provide evidence that installation of the sulfamyl moiety involves phosphoadenosine phosphosulfate (PAPS)^[21]^ and its associated enzymatic machinery, and we identify one of the two PAPS gene clusters in *S. calvus* to be essential for the biosynthesis of **1** as well as primary metabolism. Finally, we demonstrate that three genes located on the edge of the **1** BGC encode proteins appear to be required for the fluorination process; however, at this stage their specific functions are not readily assignable and therefore their roles in fluorometabolite biosynthesis remains to be determined.

## Results and Discussion

**Figure 2:**
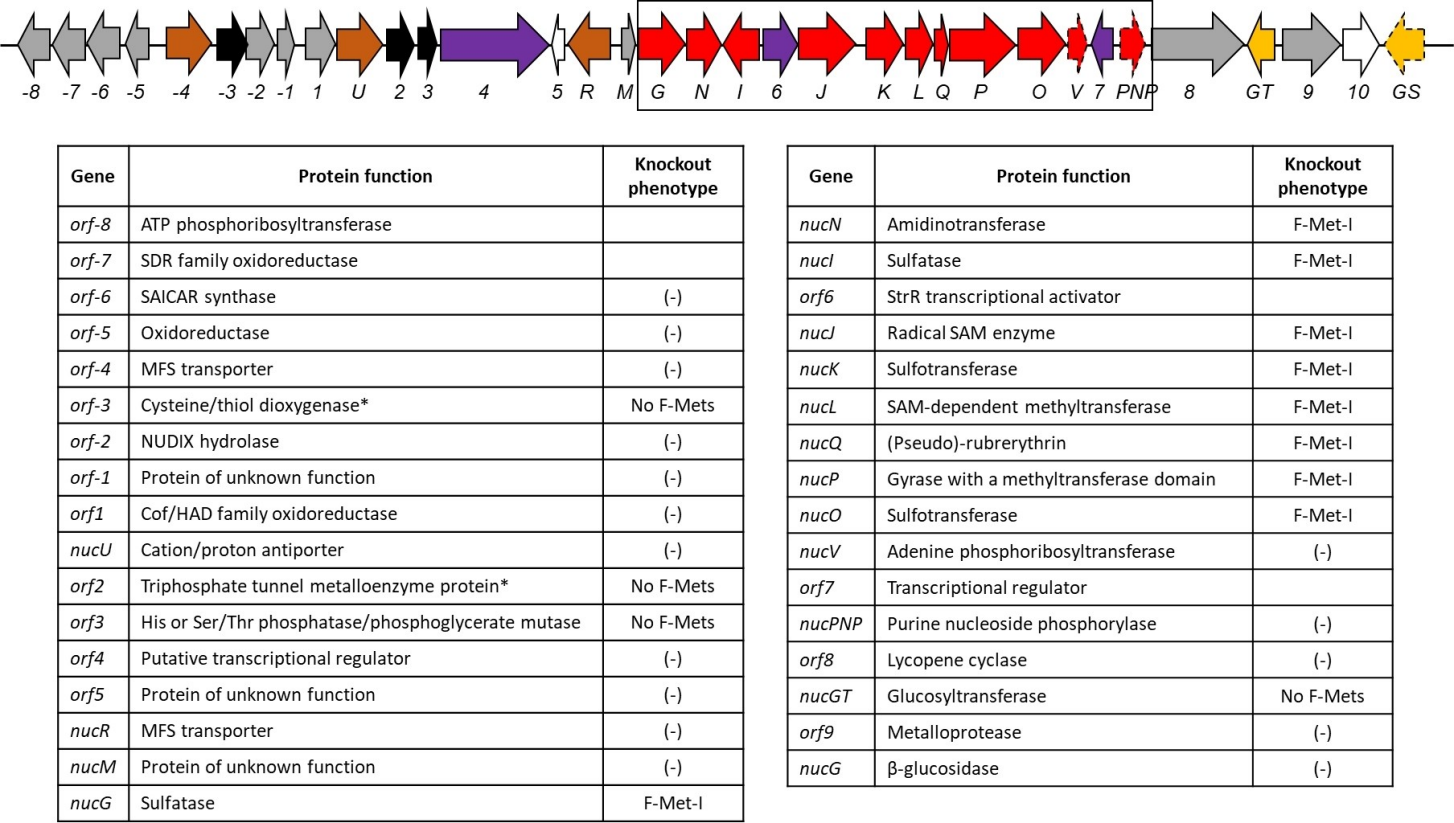
Description of the nucleocidin BGC in nucleocidin producers.[^14^, ^15^] The proposed BGCs, include a flanking region at the 5’‐end coding for enzymes with related activities (*orf−8* to *orf−5*). The numbering is relative to oxido‐reductase gene, *orf1*, which was previously implicated as the start of the BGC. Genes encoding enzymes involved in sulfamylation are shown in red, with the non‐essential ones contained in dashed lines. Proteins of unknown function involved in fluorometabolite production are encoded by genes shown in black; the genes that are not essential for nucleocidin biosynthesis are in grey. Transcriptional regulator genes are in purple, membrane transporter genes in brown, genes coding for proteins involved in glucose transfer/removal are in yellow. Empty arrows represent a gene that is absent in some of the other producers (*orf10*). An asterisk denotes function annotations that were based on AlphaFold2 structure predictions^[25]^ and comparison to known protein folds. For the knockout phenotypes, No F‐Mets indicates abolishment of fluorometabolite production, F‐Met‐1 indicates production of F‐Met‐I **2** only, symbol (−) indicates that the deletion had no effect and the fluorometabolites were produced.

The approach taken involved a systematic knockout of all the genes along the **1** BGC in *S. calvus* that, based on their annotation and predicted function, could be involved in the assembly of **1**. This included several genes encoding proteins of unknown or tentatively assigned function, with limited sequence similarity to known enzymes. Based on the previously proposed map,[^14^, ^15^] where the BGC starts with *orf1* (encoding an oxido‐reductase), the upstream genes were named accordingly – counting away from *orf1*. A revised map of the BGC and an annotation of predicted functions is shown in Figure 2. Notably, the analogous BGCs in the other producers of **1** (*S. virens*, *S. asterosporus* and *S. aureorectus*) exhibit an identical gene arrangement and composition with close to 100 % sequence identity. In one case, to accelerate the screening process, two genes (*orf2* and *orf3*) were deleted together. The outcomes of the knockouts allowed classification of the genes into three categories; a) those that have no effect on fluorometabolite production, b) those that disable sulfamylation and then c) those that disable fluorination.

Our knockout strategy involved deletion of at least half of the gene including the start codon (graphical illustration of the method is shown in Figure S1 in the Supporting Information), aiming to completely eliminate target gene expression while retaining the 3’‐region of the gene, which in many cases was likely to contain the promoter for a downstream gene. Consequently, we differentiate gene disruptions from knockouts, to account for the fact that at least part of the target gene is expressed in the former method. This approach gave results which contradict some of the outcomes recently reported by Zhu *et al*. in *S. calvus* ^[14]^ and Pasternak *et al*. in *S. virens* ^[19]^ – where four individual gene disruptions abrogated fluorometabolite biosynthesis. In our hands two of these genes have no effect on production, while knockouts of the other two result in an F‐Met‐I **2** only phenotype. The discrepancies prompted us to delete the questionable genes in *S. virens* to cross‐check the phenotypes in that strain. We have also conducted verifications of knockout genotypes including negative (knockout vector) and positive controls (genomic DNA) (Figure S2). Interestingly, we previously also generated several gene disruptions which precluded fluorometabolite production (data not shown), whereas the complete abolishment of the expression of those genes had no effect or resulted in an F‐Met‐I only phenotype. Based on these observations we conclude that the “total knockout” approach is more appropriate than the gene disruption strategy, certainly in the context of the gene clusters investigated here.

Fermentation cultures were usually harvested after 5–7 days, generally before the accumulation of nucleocidin **1** in the culture media. Importantly F‐Met‐II **3** served as a proxy for nucelocidin production– its presence confirmed that both fluorination and the sulfamylation processes were retained in the mutant strain.

### Genes that do not affect fluorometabolite production

**Figure 3:**
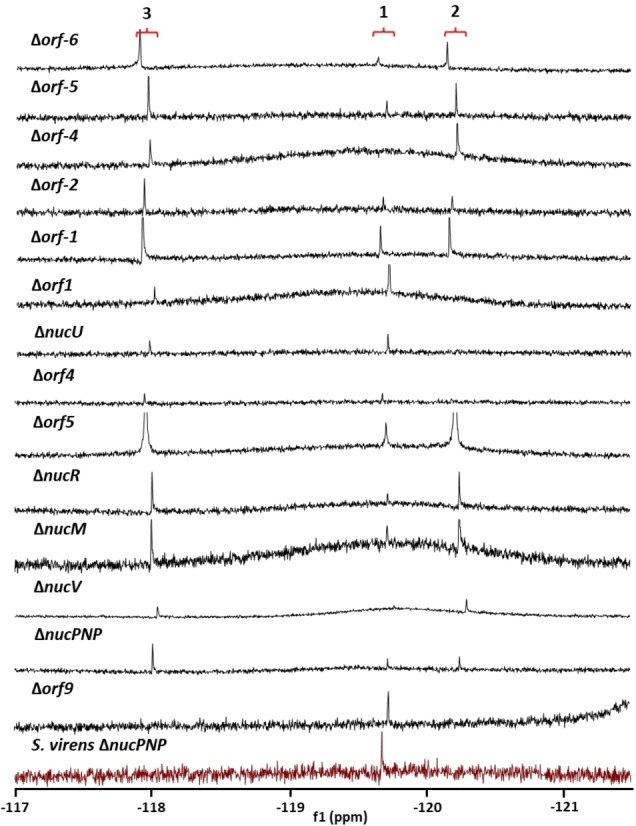
^19^F(^1^H) NMR spectra of fluorometabolite profiles from cultures of 14 gene knockouts in and around the proposed nucleocidin **1** BGC. The resultant ^19^F{^1^H} NMR signals represent either F‐Met‐II **3** (ca. −118 ppm), nucleocidin **1** (ca. −119.7 ppm) or F‐Met‐I **2**(ca. −120.3 ppm).

Systematic knockouts along the BGC identified many genes that are not critical for the biosynthesis of **1** (Figure 3). In agreement with the observation by Pasternak *et al*.,^[19]^ we found that *orf8*, encoding a putative lycopene cyclase, is not essential for fluorometabolite formation (data not shown). The list of genes irrelevant to the production of **1** includes several homologues of genes found in the BGC for (dealanyl)ascamycin **7**, **8** – *nucM*, *nucPNP*, *nucR*, *nucU* and *nucV*. Notably, *nucPNP* was previously reported to be essential for fluorometabolite biosynthesis.^[14]^ To ensure that this discrepancy is due to genetic manipulation, rather than differences between the strains used, we also deleted *nucPNP* in *S. virens* – obtaining the same wild‐type like phenotype (Figure 3, in magenta). Interestingly, *nucPNP* and *nucV* have homologues with 68 and 62 % sequence identity, respectively, in the genomes of **1** producers. NucV and its paralogue *Sc*APRT are adenine phosphoribosyltransferases which interconvert 5‐phospho‐α‐d‐ribose‐1‐diphosphate (PRPP) and adenine with adenosine‐5′‐monophosphate (AMP). These two enzymes have recently been assayed and *Sc*APRT showed greater activity, leading to the suggestion that NucV might be optimised to act on the 4‐fluororibose pathway whereas *Sc*APRT may be more relevant to primary metabolism.^[22]^ Nevertheless, partial or full redundancy may account for the observed wild‐type phenotype in Δ*nucV* and Δ*nucPNP* strains where knockouts had no evident effect on fluorometabolite biosynthesis. In these cases only ‘production’ or ‘no production’ phenotypes were determined (by ^19^F NMR) rather than the absolute fluorometabolite levels relative to wildtype or other mutants, therefore no conclusions regarding potential enzymatic redundancy are made.

### Genes required for sulfamylation

**Figure 4:**
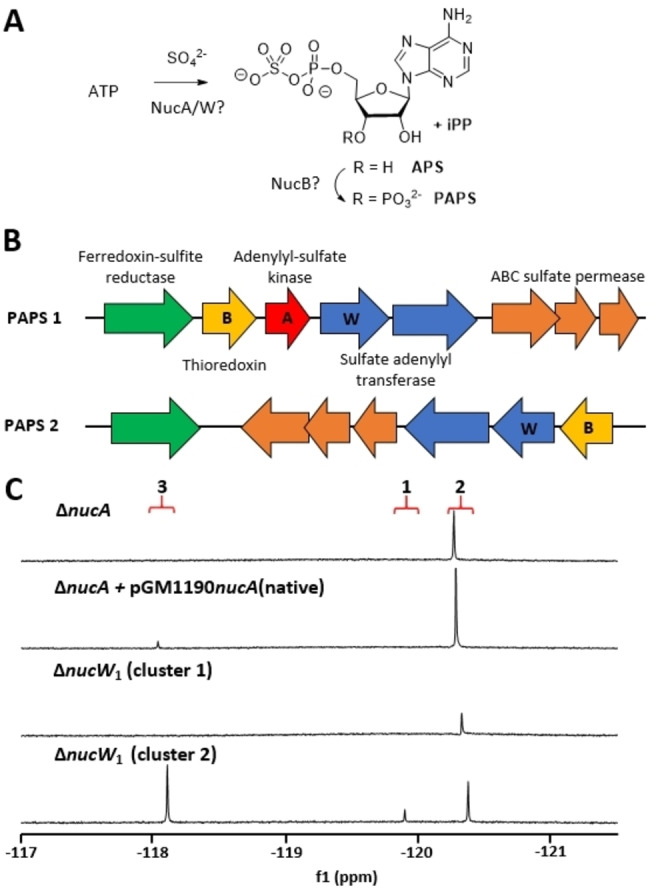
PAPS gene clusters and their involvement in nucleocidin biosynthesis. A) The PAPS pathway highlighting the steps likely to involve NucA, NucB and NucW, the enzymes were found to play a role in nucleocidin biosynthesis. B) Schematic representation of PAPS clusters in *S. calvus*; the genes indicated with letters are homologues of *acmA*, *acmB* and *acmW* present in the (dealanyl)ascamycin BGC. C) ^19^F{^1^H} NMR spectra of media extracts from cultures of *nucA* or *nucW* knockout strains; deletion of *nucA* was successfully complemented with a “native” expression plasmid. Signal assignments: F‐Met‐II **3** (ca. −118. ppm), nucleocidin **1** (ca. −119.7 ppm) and F‐Met‐I **2** (ca. −120.3 ppm).

Genes whose knockouts resulted in the production of F‐Met‐I **2**, but not F‐Met‐II **3**, were grouped as those that are involved (directly or indirectly) in sulfamyl assembly. Sulfation in biochemistry generally takes place through the activation of sulfate by ATP to generate phosphoadenosine phosphosulfate (PAPS) as a sulfate transfer agent (Figure 4A).^[21]^ In contrast to the **1** BGC in *S. calvus* and other **1**‐producing strains, the (dealanyl)ascamycin BGC in *Streptomyces* sp. JCM9888^[11]^ contains several genes encoding enzymes of the PAPS cluster, including adenylate sulfate kinase (*acmA*), thioredoxin (*acmB*) and subunit 2 of sulfate adenylyl transferase (*acmW*). This implied that the PAPS pathway may be involved in the biosynthesis of the sulfamyl moiety. Analysis of the *S. calvus* genome revealed two candidate PAPS clusters which may encode enzymes responsible for sulfate activation (Figure 4B). Each cluster encodes two subunits of sulfate adenylyltransferase, three proteins involved in sulfate transport as well as a thioredoxin and a ferredoxin‐sulfite reductase. Interestingly, only one of these clusters contains an adenylate sulfate kinase gene. For clarity and brevity, and by analogy to the *acm* genes, the adenylyl sulfate kinase and thioredoxin are referred to here as NucA and NucB, respectively, while the two subunits of adenylylsulfate transferase are termed NucW_1_ and NucW_2_. Neither cluster is located in or close to the nucleocidin **1** BGC and hence there was no indication by proximity if either play a role in the biosynthesis of **1**. However, it was anticipated that one could be involved in arming sulfate for incorporation into nucleocidin while the other (encoding the full set of PAPS enzymes, i. e., cluster 1, Figure 4B) may have a role in primary metabolism.

To verify if either PAPS cluster is involved in nucleocidin biosynthesis both *nucW*_1_ genes as well as the only *nucA* gene (cluster 1) were individually knocked out. Gene deletion within PAPS cluster 2 had no effect on fluorometabolite production, while each of the two gene knockouts within cluster 1 abolished sulfamylation as only F‐Met‐I **2** could be detected in culture media extracts (Figure 4C). An attempt to complement the knockout of the adenylyl sulfate kinase gene using an inducible pGM1190^[23]^‐based expression vector did not work; however, sulfamylation was successfully restored with a ‘native’ complementation vector containing ∼200 bp of upstream sequence in place of the thiostrepton‐inducible promoter (see the Supporting Information for construct details). Notably, both gene deletions in cluster 1 had a visibly detrimental impact on *S. calvus* growth, given that in the knockout process the proportion of double‐crossover recombinants obtained was very low in each case (<5 % of the colonies screened), and the knockout strains grew much slower than wild type both in liquid culture and on solid media (data not shown).

**Figure 5:**
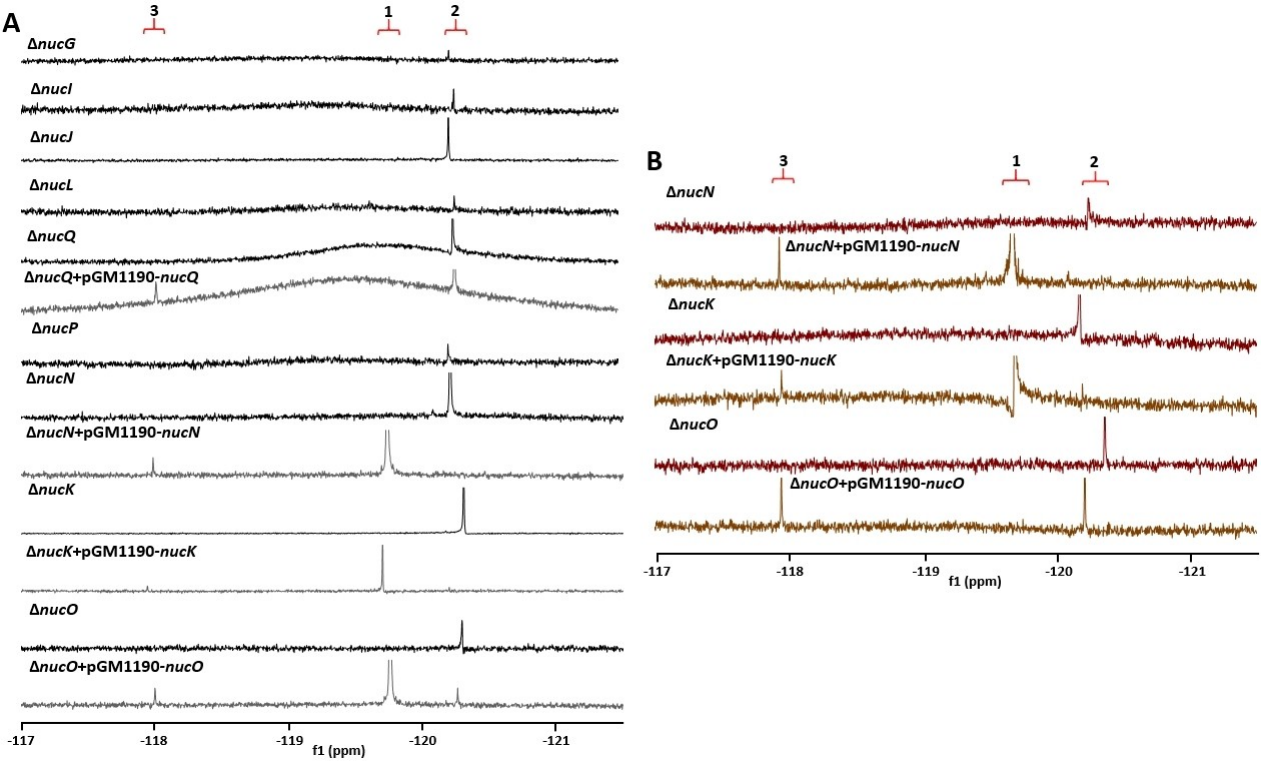
Representative ^19^F{^1^H} NMR spectra for media extracts from cultures of A) nine *S. calvus* knockout strains (black lines) along with gene complementation *in trans* (grey) and B) *S. virens* Δ*nucN*, Δ*nucK* and Δ*nucO* (magenta) together with gene complementation (brown). Sulfamylation is dependent on at least nine genes of the nucleocidin BGC. Signal assignments: F‐Met‐II **3** (ca. −118 ppm), nucleocidin **1** (ca. −119.7 ppm) and F‐Met‐I **2** (ca. −120.3 ppm).

Of all the genes deleted within the **1** BGC, nine were found to be associated with the installation of the sulfamyl moiety, alongside the PAPS genes (Figure 5A). Three of these (*nucI, nucG* and *nucJ*) were recently reported by Pasternak *et al*.,^[19]^ as essential for the production of **3** and **1**. The present study extends the list of genes essential for sulfamylation to include *nucK*, *nucL*, *nucN*, *nucO*, *nucQ* and *nucP*, none of which have paralogues in the genomes of nucleocidin **1** producers. Therefore at least eleven enzymes, encoded in two different gene clusters, are essential for sulfamyl group biosynthesis. Notably, and in contradiction, disruptions of *nucK*, *nucN* and *nucO* were recently reported to abolish fluorometabolite production in *S. virens*,^[19]^ while the disruption of *nucK* homologue, *acmK*, previously abrogated production of (dealanyl)ascamycin **7**, **8** in *Streptomyces* sp. JCM9888.^[11]^ To further validate our results we generated knockouts of these three genes in *S. virens*. In line with the results from *S. calvus*, *S. virens* Δ*nucK*, Δ*nucN* and Δ*nucO* all produced F‐Met‐I **2** (Figure 5B), demonstrating that the proteins encoded by these three genes are involved in the sulfamylation process. Furthermore, we successfully complemented these knockout phenotypes *in trans* in both strains.

NucK and NucO both annotate as sulfotransferases, although only ∼40 % of the NucK sequence overlaps with NucO (35 % sequence identity for that region). In this respect the annotation of the NucO sulfotransferase domain seems tentative, as its detection by various domain prediction servers is associated with a relatively low confidence value – for example, Pfam^[24]^ annotates region 226–310 as “Sulfotransfer_3” with a low *E* value (2.6×10^−8^). For comparison, the *E* values for “Sulfotransfer_1” (5.2×10^−13^) and “Sulfotransfer_3” (3.7×10^−39^) domain annotations in NucK are considerably lower and thus indicating strong similarity to known domains. A structure prediction obtained for NucO from AlphaFold2^[25]^ was compared to other known structures using the DALI server;^[26]^ CurM sulfotransferase (PDB ID: 4GBM), involved in curacin A biosynthesis, and an olefin synthase sulfotransferase (PDB ID: 4GOX) were identified as the closest fold homologues.^[27]^ Both of these enzymes utilise sulfonation as a means to activate their respective substrates for catalysis. It is therefore plausible that NucK and NucO both possess sulfotransferase activity but have different roles in nucleocidin **1** biosynthesis. Given its annotation as an amidinotransferase, NucN may be involved in installing the amino group of the sulfamyl moiety of nucleocidin, although this remains tentative. NucL, NucQ and NucP are predicted to be a SAM‐dependent methyltransferase, a rubrerythrin and a gyrase with a C‐terminal methyltransferase domain, respectively. Rubrerythrins are non‐heme iron proteins involved in oxidative stress responses.^[28]^ Despite its annotation, NucQ lacks two of the six critical residues generally associated with iron binding as well as the short C‐terminal rubredoxin domain (Figure S3). This protein is therefore referred to here as a pseudo‐rubrerythrin as it remains to be shown whether it uses iron for catalysis. The sulfamylation‐deficient phenotype of Δ*nucQ* cells was complemented with an inducible expression plasmid containing the functional copy of this gene (Figure 5A).

### Genes that block fluorometabolite production

We previously reported *nucGT* as essential for nucleocidin **1** biosynthesis, as no fluorometabolites could be detected in the media extracts of Δ*nucGT* cells.^[17]^ However, β‐glucosylation activity of NucGT may be required for export and/or detoxification of **1** and its cognate metabolites, rather than the fluorination process directly. In fact, LC–MS analysis of the Δ*nucGT* strain did not identify any **1**‐associated metabolites, including the sulfamylated de‐fluorocompound **9**, in the media extracts.^[20]^ Lack of glucosylation activity therefore appears to affect the pathway more globally, most likely precluding metabolite export out of the cell.

**Figure 6:**
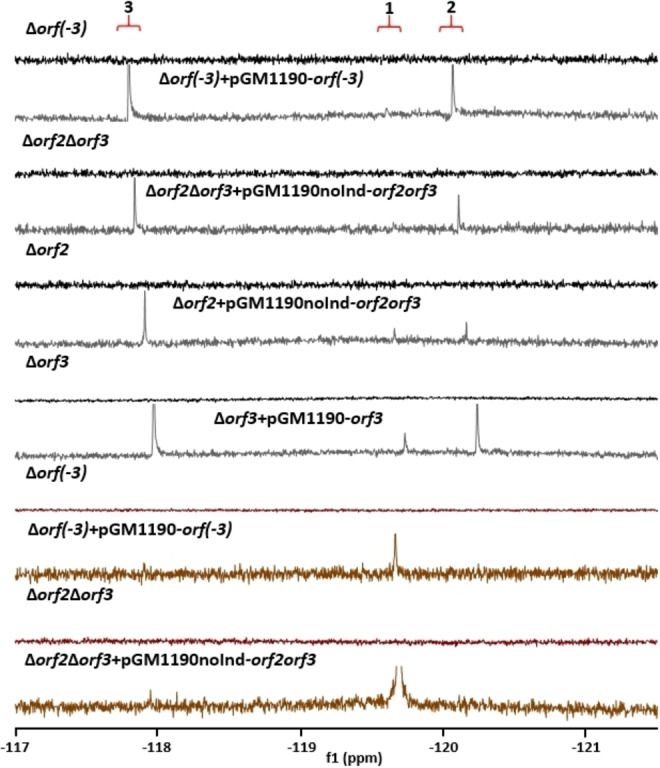
Representative ^19^F{^1^H} NMR spectra for media extracts from cultures of knockouts and complementation experiments with *orf(−3)*, *orf2* and *orf3*. A) Δ*orf(−3)*, Δ*orf2*Δ*orf3*, Δ*orf2* and Δ*orf3* (black lines) along with gene complementation *in trans* (grey) and B) *S. virens* Δ*orf(−3)* and Δ*orf2*Δ*orf3* (magenta) together with gene complementation (brown). Signal assignments: F‐Met‐II **3** (ca. −118 ppm), nucleocidin **1** (ca. −119.7 ppm) and F‐Met‐I **2** (ca. −120.3 ppm).

Three genes were identified in our knockout screen which appear to be essential for fluorination. These are *orf(−3)*, *orf2* and *orf3*, located upstream of the sulfamylation gene cluster. The ^19^F{^1^H) NMR profiles of media extracts from the corresponding knockout strains are illustrated in Figure 6. To accelerate the screening process, *orf2* and *orf3* were initially deleted together and this knockout abolished fluorometabolite production. The double knockout was then successfully complemented *in trans* with both “native” and thiostrepton‐inducible plasmids. The inducible vector was constructed so that the genes are under the control of one *tipA* promoter, which would result in a bicistronic mRNA with two ribosome binding sites, and *orf2* was placed after *orf3* to prevent potential early transcription termination due to a natural terminator – which could be located in the *orf3* sequence. We then proceeded to individual gene knockouts, and in each case the same phenotype was obtained, implying that the products of both genes are essential for the fluorination process. Interestingly, fluorometabolite production was readily restored in Δ*orf(−3)* and Δ*orf3* cells with both “native” and inducible pGM1190‐based plasmids, while *orf2* deletion could only be complemented with vectors containing both *orf2* and *orf3* (either inducible or “native”), suggesting a genetic linkage between these neighbouring genes or a functional co‐dependence between the enzymes they encode. To further validate these results, *orf(−3)*, as well as *orf2* and *orf3* in tandem, were deleted in *S. virens* and this produced the same fluorination‐deficient phenotype. In each case these deletions were successfully complemented *in trans* in *S. virens*, restoring fluorometabolite production (Figure 6). Importantly, LC–MS analysis revealed that the de‐fluorinated co‐metabolites **9** and **10** were present in the media extracts from cultures of Δ*orf(−3)*, Δ*orf2*Δ*orf3* and Δ*orf2* (Figures S4–S13). These findings clearly indicate that all aspects of the biosynthesis – including sulfamylation, glucosylation and export – are in place in the knockout strains, except the fluorination activity.

**Figure 7:**
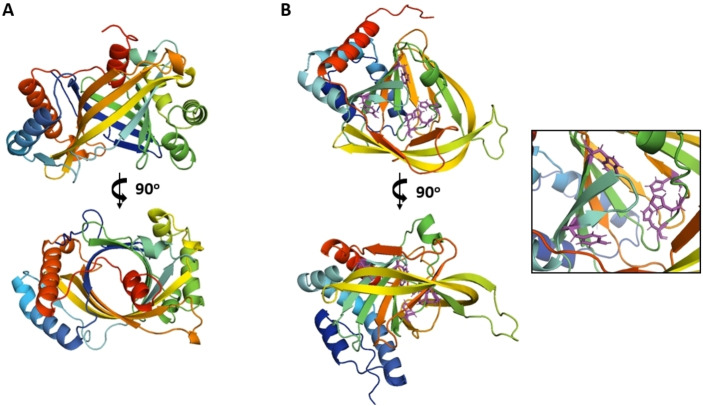
AlphaFold2^[25]^ structure predictions for Orf2 and Orf(−3). A) Putative structure of Orf2 revealing a similarity to the triphosphate tunnel metalloenzyme family. B) Putative structure of Orf(−3), which shares fold similarity with cysteine/thiol dioxygenases. The framed inset shows a close up of the putative active site, modelled on the closest homologues, including three histidines (His115, His117 and His200), a tyrosine (Tyr73) and a tryptophan (Trp100).

Orf3 annotates as a histidine phosphatase or phosphoglycerate mutase; however, no putative function could be ascribed to Orf2 or Orf(−3) based on their sequences. AlphaFold2^[25]^ was employed to predict the structures of the latter two proteins, in an attempt to relate them to known protein folds. The predictions were generated with high confidence, as judged by final assessment parameters, and the resulting PDB files were submitted to the DALI server^[26]^ for fold comparison. The predicted structure models are shown in Figure 7 and the graphical assessments of prediction quality in Figures S14 and S15.

Orf2 exhibits highest fold similarity to the family of triphosphate tunnel metalloenzymes (TTM). These proteins form a closed soluble β‐barrel, with an active site present on one end of the tunnel.^[29]^ TTMs hydrolyse various organophosphates with a preference for triphosphates, and normally two divalent metal cations are involved in catalysis. Orf(−3) appears to be structurally related to cysteine dioxygenase, non‐heme iron enzymes which generate sulfinic acid residues by addition of oxygen to the sulfur of cysteine.^[30]^ It remains to be determined how these enzymes contribute to enzymatic fluorination.

## Conclusions

In summary, an exhaustive knockout screen has been performed along the biosynthetic gene cluster associated with nucleocidin **1** biosynthesis in *S. calvus*, with additional confirmatory knockouts conducted in *S. virens*. Furthermore, two remote gene clusters encoding proteins of the PAPS pathway were probed for their involvement in **1** biosynthesis. The gene‐deletion strategy employed involved complete elimination of expression of the target genes (or truncation of the gene products to just a few amino acids), rather than gene disruption, and this led to a number of outcomes that differed from those recently reported elsewhere.[^14^, ^19^] These observations were cross‐validated by generating knockouts in both *S. calvus* and *S. virens* and successfully complementing the contentious deletions.

The study identified 14 genes located in or on the periphery of the proposed **1** BGC that appear to be non‐essential or not involved in fluorometabolite biosynthesis. Unexpectedly at least 11 genes were found to be involved in the installation of the sulfamyl moiety on the ribosyl ring. Two of these genes (*nucA* and *nucW*_1_) form part of PAPS cluster 1, which is associated with sulfate activation, and the other nine genes are located in the nucleocidin **1** BGC, forming a sulfamylation sub‐cluster. Most of the genes within this subcluster were already annotated as encoding sulfate‐processing enzymes, consistent with these experimental outcomes. Notably, the process of knocking out the two genes in PAPS cluster 1 resulted in a proportionally low number of double‐crossover recombinants, and the deletions had a visibly detrimental impact on cell growth, thus suggesting that the PAPS machinery encoded by this cluster also plays a role in primary metabolism. This is further supported by the presence of the adenylyl sulfate kinase gene in cluster 1, but not in cluster 2. The outcomes do not shed any light on how and when the amine substituent of the sulfamyl moiety is incorporated, or what roles the remaining enzymes encoded in nucleocidin BGC might play.

Three genes were discovered on the periphery of the sulfamylation subcluster that appear critical for fluorination, as their deletion abrogated the production of fluorometabolites in both *S. calvus* and *S. virens*. In each case, complementation *in trans* restored fluorometabolite production. The media extracts from cultures of *S. calvus* Δ*orf−3*, Δ*orf2* and Δ*orf3* strains contained defluoro‐sulfamylated and defluoro‐glucosylated co‐metabolites **9**–**11**, thus demonstrating the overall integrity of the biosynthetic pathway and strongly suggesting that these three genes play a role in fluorination biochemistry, although their activities and roles remain unknown. Taken together these insights from molecular biology should inform biochemical experiments aimed at uncovering the incorporation of the fluorine and sulfamyl moieties in nucleocidin **1**.

## Experimental Section

Full details are available in the Supporting Information.

**Molecular biology and gene knockout procedures**: Both pGM1190 (expression/complementation vector) and pKC1139^[31]^ (knockout vector) were linearised by digestion with BamHI followed by gel extraction. To generate the pKC1139‐based knockout vectors the left and right flanking regions (“arms”, ∼1500–2200 bp each) of the target gene were amplified from *S. calvus* genomic DNA. The overview of knockout plasmid design and the knockout strategy is shown diagrammatically in Figure S1. For the construction of pGM1190‐based plasmids for complementation the target genes were amplified from *S. calvus* genomic DNA and inserted in a way that resulted in the presence or absence of a 6His‐tag on the gene product. In vectors designed for thiostrepton‐inducible gene expression the gene of interest was inserted downstream of the *tipA* promoter while in the uninducible, “native” complementation plasmids (termed pGM1190noInd) the *tipA* promoter was replaced with ∼200 bp of the sequence found upstream of the target gene.

Purified PCR products were inserted into target vectors using NEBuilder HiFi assembly kit (New England Biolabs) and the reactions were transformed into *Escherichia coli* DH10β (Thermo Scientific). Resulting colonies were screened by PCR using appropriate primer pairs and candidate plasmids were extracted, verified by sequencing and transformed into *E. coli* ET12567/pUZ8002. The plasmids were transferred into *Streptomyces* cells by conjugation and in the case of knockout vectors the conjugants underwent single‐ and double‐crossover events, with colonies propagated through several generations to promote plasmid loss. Colonies that lost antibiotic resistance were screened by PCR and those showing the knockout genotype were subjected to additional verifications using different primer pairs.

**Fermentation and gene knockout complementation**: Cells were grown in tryptic soy broth, supplemented with 50 μg mL^−1^ apramycin where necessary, at 28 °C with shaking for 2–4 days. Once mycelium was dense a preculture (2 mL) was used to inoculate the fermentation broth (100 mL)^[17]^ in a 500 mL conical flask. On the day of harvest (typically between day 4 and 8) the cells were separated from the media by centrifugation (5000 *g* for 10 min). Between 1–2 flasks were normally used per sample extract. Complementation of gene knockouts involved a pGM1190 plasmid that contained the relevant gene under the control of either a thiostrepton‐inducible or a native promoter as described above. Both complementation approaches required the presence of apramycin in the culture medium, with a fresh dose added after 5 days of fermentation. Cultures containing the inducible plasmids were supplemented with thiostrepton daily from the day after inoculation, to a final concentration of 2–20 mg L^−1^.

**Extraction and analysis of (fluoro)metabolites**: Metabolites were isolated from fermentation culture media by addition of 20 % total volume of butan‐1‐ol (Fisher Scientific) and separation of the organic phase. After solvent evaporation the dried extracts were resuspended in water (∼0.7 mL) containing 10 % D_2_O and 50 mM MgCl_2_ and submitted for ^19^F{^1^H} NMR analysis. Extracts showing no fluorometabolites, as evident from the NMR analysis, were partially purified and submitted for LC–MS analysis.

## Conflict of interest

The authors declare no conflict of interest.

1

## Supporting information

As a service to our authors and readers, this journal provides supporting information supplied by the authors. Such materials are peer reviewed and may be re‐organized for online delivery, but are not copy‐edited or typeset. Technical support issues arising from supporting information (other than missing files) should be addressed to the authors.

Supporting Information

## Data Availability

The data that support the findings of this study are available in the supplementary material of this article.
