# Supplementary material for: Identification of Genes Essential for Fluorination and Sulfamylation within the Nucleocidin Gene Clusters of Streptomyces calvus and Streptomyces virens
Source: Chembiochem. 2023 Jan 26;24(5):e202200684. doi: 10.1002/cbic.202200684 (PMC10946740; doi:10.1002/cbic.202200684)
Supplement: Supplementary file 1 — Supporting Information [file CBIC-24-0-s001.pdf]

ChemBioChem

Supporting Information

Identification of Genes Essential for Fluorination and Sulfamylation within the Nucleocidin Gene Clusters of *Streptomyces calvus* and *Streptomyces virens*

Marta Wojnowska, Xuan Feng, Yawen Chen, Hai Deng, and David O'Hagan*

Index **Page**

1. General experimental procedures 2
2. Supplementary tables 6
3. Supplementary figures 15
4. References 29

1. General experimental procedures

Room temperature refers to 18-25 °C. All microbiological work was carried out in a Gallenkamp laminar flow hood. Glassware and consumables for biological operations were sterilised by autoclaving or wiping with 70% ethanol before using. Sterilised consumables were used as supplied. Media were sterilised by autoclaving. Cell cultures were incubated in a temperature-controlled orbital incubator (New Brunswick Scientific). Centrifugation of 20 mL to 1 L was processed by Beckman Avanti centrifuge. A ThermoFisher Heraeus Fresco 21 benchtop refrigerated centrifuge was used for micro-centrifugation. All evaporations and concentrations were performed under reduced pressure (*in vacuo*) by Büchi Rotavapor R-200.

^{19}F {^{1}H} NMR analysis

^{19}F NMR and ^{19}F {^{1}H} NMR spectra were recorded at 298 K on Bruker Advance II 400, Advance III HD 500 instruments using $CFCl_3$ as an external reference. Chemical shifts are reported in parts per million (ppm) and coupling constants (*J*) are reported in Hertz (Hz).

LC-MS analysis

Extracts from culture media were freeze dried, resuspended in 50% acetonitrile to about half the original volume and centrifuged at maximum speed for 10 min to remove particulates. These samples were analysed at the Mass Spectrometry Facility at the University of St Andrews using ThermoFisher Xcalibur Orbitrap instrument in positive ion mode. Due to low abundance of metabolites some samples were partially purified by HPLC; majority of the acetonitrile/water elution fractions were collected and after removing the solvent the dry extracts were resuspended in water. High resolution electrospray ionization spectra were acquired on a Bruker MaXis II ESI-Q-TOF-MS connected to a Dionex 3000 RS UHPLC fitted with an ACE C4-300 RP column (100 x 2.1 mm, 5 µm, 30°C). The metabolites were eluted with a linear gradient of 5–100% MeCN containing 0.1% formic acid over 30 min. The mass spectrometer was operated in positive ion mode with a scan range of 200–3000 m/z. Source conditions: end plate offset at −500 V; capillary at −4500 V; nebulizer gas (N2) at 1.8 bar; dry gas (N2) at 9.0 L min−1; dry temperature at 200 °C. Ion transfer conditions: ion funnel RF at 400 Vpp; multiple RF at 200 Vpp; quadrupole low mass at 200 m/z; collision energy at 8.0 eV; collision RF at 2000 Vpp; transfer time at 110.0 µs; pre-pulse storage time at 10.0 µs. MS data were analysed using Bruker DataAnalysis.

Plasmid design and construction

All the primers were synthesised by Merck. Plasmids and, where necessary, PCR products were sequenced by Eurofins Genomics. Table S1 shows the list of plasmids used and/or generated in this study while Tables S2-4 contain primers that were used for PCR and sequencing. Amplification of genes intended for cloning was conducted with Q5 High Fidelity polymerase (NEB) supplemented with 7.5% DMSO while Ultra polymerase (PCR Biosystems) with 2.5% DMSO was employed for colony screening and general verifications. Both polymerases were used according to the manufacturers' protocols with the usual annealing temperatures of 59-62°C (Ultra) and 62-65°C (Q5). PCR products used in cloning were gel extracted and then subjected to an additional DNA purification using Monarch DNA kits (NEB). NEBuilder HiFi assembly master mix (NEB) was used to insert DNA fragments into the target vectors, following manufacturer's protocol. The assembly reactions were diluted 4-fold with distilled water and 15μl were then transformed into 100μl chemically competent *E. coli* DH10β (Thermo Scientific). The resulting colonies were screened by PCR using different combinations of gene-specific cloning and verification primers (listed in Table S1), as well as vector-specific primers; for the knockout vectors pKC1139seqF (5'-GCCAGGGTTTTCCCAGTCACGACG-3') and pKC1139seqR (5'-CCGGCTCGTATGTTGTGTGGAATTGTGAG-3') while for the complementation/expression vectors pGM1190seqF (5'-GGAACGTCCGGGCTTGCACCTCACGTC-3') and pGM1190seqR (5'-CAAAACTTTAGATCTGGGTTAGCAGCCGG-3').

The inserts for "native" vectors were generated by amplifying the gene(s) of interest along with ~200 bp of upstream sequence. The 5' end of these inserts were complementary to a sequence upstream of *tipA* and the 3' end contained the natural stop codon, so that the inducible promoter was replaced with a native one, and no affinity tag was present on the resulting gene product. For screening and sequencing of this type of plasmid pGM1190(noInd)F primer (5'- GGCGCGACAAGTTGCTGCGATTCTCACC-3') was employed in place of pGM1190seqF.

Double-expression orf2+orf3 plasmid was constructed using pGM1190-Orf3his as the backbone and pGM1190-Orf2his as a template for insert amplification. The strategy exploited a unique cleavage site – BglII cuts right after the *his6* region but before the terminator sequence. The RBS-*orf2-his6* amplicon was inserted into BglII-digested pGM1190-Orf3his plasmid using the NEBuilder assembly kit, resulting in a tandem-expression construct which would be transcribed as a bicistronic mRNA. Gene order was deliberately inverted, so that *orf2* is after *orf3*, to ensure the expression is regulated by vector-borne genetic elements rather than the native ones. Screening and sequencing employed primers BglIIseqF (5'-

CACTGAGATCCGGCTGCTAACCCAGATC-3’) and BglIIseqR (5’-GTTCCTTTCTATTCTCACTCCGCTGAAACTG-3’).

Most gene knockouts involved the removal of between half and two-thirds of the target gene, including the start codon. In three cases the start codon was mutated due to a partial overlap of the target gene with the upstream gene; for *orf2* deletion the last four nucleotides of nucU were changed from **GTG**A into GTAG, for *nucK* knockout the last four nucleotides of nucJ were mutated from **ATG**A into CTAA and for *nucP* knockout the seven base pair overlap with preceding nucQ was changed from **ATG**AGTGA into AGGAATGA. In the case of *orf(-2)* knockout the start codon was retained due to 17bp overlap with the upstream gene, however a stop codon was inserted in frame immediately after the starting sequence. The *nucO* deletion was accidentally made so that the four nucleotides overlapping with the preceding *nucP* gene were joined with the terminal 24bp of *nucO*, resulting in a frame shift which would eliminate the natural stop codon. However, the generation of a long nonsense polypeptide is probably prevented by the transcription terminator, most likely located in the sequence of the downstream gene (*nucV*). The intermediate phenotype conferred by this gene knockout and the fact that was readily complemented *in trans* suggests that despite the mistake, the phenotype is likely to be real and the frameshift did not affect the cells in a negative way. One other unintended change occurred in the process of *orf3* knockout, where the third base of the natural *orf2* stop codon was accidentally removed and the resulting *orf2* gene product in *Δorf3* cells contained additional two amino acids at the C terminus (Trp, Glu). However, as the difference was marginal and the phenotype associated with the absence of *orf3* was readily complemented the sequence of *orf2* was not fixed in this knockout strain.

Plasmid conjugation into *Streptomyces* cells

Knockout and complementation plasmids were transformed into *E. coli* ET12567/pUZ8002, which are capable of plasmid conjugation into *Streptomyces* cells. Cell pellets from overnight cultures of these cells were washed twice with LB medium and the mycelium of the target *Streptomyces* was harvested and resuspended in LB. Different *E. coli* ETU to *Streptomyces* ratios were used for conjugation, and the cell mixtures were plated onto ISP4 agar plates. After ~20 hours of incubation at 30°C the plates were overlaid with 1 ml LB containing 0.5 mg nalidixic acid and 1.25 mg apramycin, allowed to dry and incubated for a few days until the appearance of streptomycete colonies.

Gene knockout procedure

Figure S1 shows an overview of the gene deletion approach employed here. *Streptomyces* cells harbouring the knockout vector were plated onto ISP4 agar containing 50 μg ml^{-1} apramycin and incubated at 37°C overnight. The colonies were then re-plated onto ISP4 agar

without antibiotic and incubated at 30°C for 1-2 days to allow for the double crossover and plasmid loss; the process was repeated several times after which the colonies were replica-plated on plates with and without antibiotic to test for plasmid presence. Colonies that lost apramycin resistance were screened using gene-specific verification primers to verify their knockout status. Additional verification primers were sometimes employed if the PCR results were not clear enough.

2. Supplementary tables

Table S1. List of plasmids used in this study.

PLASMID	INDUCIBLE	DESCRIPTION	SOURCE
pKC1139	NO	Vector for gene knockouts in *Streptomyces*	[1]
pGM1190	YES	Vector for thiostrepton-inducible gene expression in *Streptomyces*	[2]
pKC-nucA	NO	Knockout vector for nucA	This work
pKC-nucW$_1$ (c1)	NO	Knockout vector for nucW$_1$ in PAPS cluster 1	This work
pKC-nucW$_1$ (c2)	NO	Knockout vector for nucW$_1$ in PAPS cluster 2	This work
pKC-orf(-6)	NO	Knockout vector for *orf(-6)*	This work
pKC-orf(-5)	NO	Knockout vector for *orf(-5)*	This work
pKC-orf(-4)	NO	Knockout vector for *orf(-4)*	This work
pKC-orf(-3)	NO	Knockout vector for *orf(-3)*	This work
pKC-orf(-2)	NO	Knockout vector for *orf(-2)*	This work
pKC-orf(-1)	NO	Knockout vector for *orf(-1)*	This work
pKC-orf1	NO	Knockout vector for *orf1*	This work
pKC-nucU	NO	Knockout vector for *nucU*	This work
pKC-orf2	NO	Knockout vector for *orf2*	This work
pKC-orf3	NO	Knockout vector for *orf3*	This work
pKC-orf2orf3	NO	Knockout vector for *orf2* and *orf3* together	This work
pKC-orf4	NO	Knockout vector for *orf4*	This work
pKC-orf5	NO	Knockout vector for *orf5*	This work
pKC-nucR	NO	Knockout vector for *nucR*	This work
pKC-nucM	NO	Knockout vector for *nucM*	This work
pKC-nucG	NO	Knockout vector for *nucG*	This work
pKC-nucN	NO	Knockout vector for *nucN*	This work
pKC-nucI	NO	Knockout vector for *nucI*	This work
pKC-nucJ	NO	Knockout vector for *nucJ*	This work
pKC-nucK	NO	Knockout vector for *nucK*	This work
pKC-nucL	NO	Knockout vector for *nucL*	This work
pKC-nucQ	NO	Knockout vector for *nucQ*	This work
pKC-nucP	NO	Knockout vector for *nucP*	This work
pKC-nucO	NO	Knockout vector for *nucO*	This work
pKC-nucV	NO	Knockout vector for *nucV*	This work
pKC-nucPNP	NO	Knockout vector for *nucPNP*	This work
pKC-orf9	NO	Knockout vector for *orf9*	This work
pGM-nucA	YES	Expression vector for NucA	This work
pGM(noInd)-nucA	NO	Native expression vector for NucA	This work
pGM-orf(-3)his	YES	Expression vector for 6His-tagged Orf(-3)	This work
pGM(noInd)-orf(-3)	NO	Native expression vector for Orf(-3)	This work
pGM-orf2his	YES	Expression vector for 6His-tagged Orf2	This work
pGM(noInd)-orf2	NO	Native expression vector for Orf2	This work
pGM(noInd)-nucU+orf2	NO	Native expression vector for NucU and Orf2	This work
pGM(noInd)-orf2+orf3	NO	Native expression vector for Orf2 and Orf3	This work
pGM-orf3his	YES	Expression vector for 6His-tagged Orf3	This work
pGM(noInd)-orf3	NO	Native expression vector for Orf3	This work
pGM-orf2his+orf3his	YES	Expression vector for Orf3-6His and Orf2-6His; the corresponding genes share the *tipA* promoter and the terminator but have individual ribosome binding sites	This work
pGM-nucN	YES	Expression vector for NucN	This work

pGM-nucK	YES	Expression vector for NucK	This work
pGM-nucQhis	YES	Expression vector for 6His-tagged NucQ	This work
pGM-nucOhis	YES	Expression vector for 6His-tagged NucO	This work

Table S2. List of primers used to construct left (LA) and right arms (RA) flanking the target genes.

TARGET GENE	PRIMER NAME	PRIMER SEQUENCE
nucA	nucA_LA_F	GACGGCCAGTGCCAAGCTTCATCACCGACCGGCAGAACGTCC
	nucA_LA_R	GGTGAGACCGGTGAGTTCGGGTCAGCCGTGCAGTCCGCACTCG
	nucA_RA_F	CGAACTCACCGGTCTCACCGGTGTGGACG
	nucA_RA_R	GACATGATTACGAATTCCACGGTGGCCTCGACGTCCTGCGTG
nucW$_1$ (c1)	nucW1_LA_F	GACGGCCAGTGCCAAGCTTCACCTTCGGCAAGCGCTTCTGCGTG
	nucW1_LA_R	CGGTGATCGTGGTCGTCCGTCTAGAAGTACCCCTCGCGCTTG
	nucW1_RA_F	GGACGACCACGATCACCGGGATCGACCTG
	nucW1_RA_R	GACATGATTACGAATTCGCAGGGCGAGTTCGATGTTCTTGCC
nucW$_1$ (c2)	nucW2_LA_F	GACGGCCAGTGCCAAGCTTGAGTTCCTCGTGCAGGACGTCCCG
	nucW2_LA_R	GCGCGAAGGATACTTCTGAGGTCATCAGCGGCATCGACCTGCTG
	nucW2_RA_F	CAGAAGTATCCTTCGCGCTTGCGGTCCTCC
	nucW2_RA_R	GACATGATTACGAATTCGCTTGGTGAACTCGTCGGTGTTGACG
orf(-6)	orf-6_ LA_F	GACGGCCAGTGCCAAGCTTGAACGCTGATCATCAGCGCGAAGC
	orf-6_ LA_R	GTCCCGGGTACTTGATGAGCACCTGGAATGTCATGGGCTCGAG
	orf-6_ RA_F	CGCTCATCAAGTACCCGGGACTGTTCG
	orf-6_ RA_R	GACATGATTACGAATTCGCTCGGTGTGACAGGGTTCGACACC
orf(-5)	orf-5_ LA_F	GACGGCCAGTGCCAAGCTTCTGGCGGTCGCTCGTCGCCTGCGG
	orf-5_ LA_R	GCGAGTTCACCGATCATCGTTCACGGTGCGGATGTTCGAGAG
	orf-5_ RA_F	GGATGATCGGTGAACTCGCCGTCCACG
	orf-5_ RA_R	GACATGATTACGAATTCGGCCCGTTTCGACGACGTCGATCAC
orf(-4)	orf-4_ LA_F	GACGGCCAGTGCCAAGCTTCGTTCCTCGATGAGGAGCGTCTCGG
	orf-4_ LA_R	GTACGCCGCCGTGCCGAGGGATTCCCTACTGATGAGTATTTCG
	orf-4_ RA_F	CTGATGCTCCTCGGCACGGCGGCGTAC
	orf-4_ RA_R	GACATGATTACGAATTCGCGTACTCGGCGGTGAACAGGTACG
orf(-3)	orf-3_ LA_F	GACGGCCAGTGCCAAGCTTGCCCAGACCGAGCACCGTCAGCGC
	orf-3_ LA_R	GTACACGTTGGAGAAGTCGCGCACCTTCTCCTGTAACTGGTCC
	orf-3_ RA_F	CGCGACTTCTCCAACGTGTACAACACCTC
	orf-3_ RA_R	GACATGATTACGAATTCGCCAGGGCGACCACCTCTTCGCAGG
orf(-2)	orf-2_ LA_F	GACGGCCAGTGCCAAGCTTCATGACCACGGATTCCGATTCATC
	orf-2_ LA_R	GTTCGACCACCTGGATGTCTAGTCAGGCGGTCCGGCCATCGCCC
	orf-2_ RA_F	GGACATCCAGGTGGTCGAACTGCCCCTGG
	orf-2_ RA_R	GACATGATTACGAATTCGCGAACGTGGCCACGCCGTCGAGG
orf(-1)	orf-1_ LA_F	GACGGCCAGTGCCAAGCTTCCTGGGCACGTTCGCCGGCAGTTC
	orf-1_ LA_R	CCGAAGGTGAGCCGGACCGGTGTCCTTTCCGACGTGTCGTCC
	orf-1_ RA_F	CACGGGTCCGGCTCACCTTCGGCGACG
	orf-1_ RA_R	GACATGATTACGAATTCGGGATGCCCAGATCGAGCATGATTC
orf1	orf1_ LA_F	GACGGCCAGTGCCAAGCTTGAGTTACGCGACGAGCTCATCCATG
	orf1_ LA_R	GTGGAGCCGGTGCGTCCAGCCTCCAGGGTTCTCGGATGCCACC
	orf1_ RA_F	CTGGACGCACCGGCTCCACGACACGC
	orf1_ RA_R	GACATGATTACGAATTCGCCTTCTCCTCGCCGTCGAACAC
nucU	nucU_LA_F	GACGGCCAGTGCCAAGCTTGCCATCGAGCTCGTACAGGGCGAGG
	nucU_LA_R	GAACAGCAGCCGGTACGCCCGGCCAGCGCGTCGTCGAACGTGG
	nucU_RA_F	GGCGTACCGGCTGCTGTTCGTGCTGGTGC
	nucU_RA_R	GACATGATTACGAATTCGGTGGTGGCCGGATCGGTGCTACTCC
orf2	orf2_ LA_F	GACGGCCAGTGCCAAGCTTGACGGCCTGCGAAGAGGTGGTCG
	orf2_ LA_R	GTCGAGCCAGACGAAGAACCTACTTCAGGTGCTCCTTCGGGTGAG
	orf2_ RA_F	CGTTCTTCGTCTGGCTCGACGAGCGGGAC
	orf2_ RA_R	GACATGATTACGAATTCGCGGTGCCGGCGTGTTCCTTGCGG
orf3	orf3_ LA_F	GACGGCCAGTGCCAAGCTTGTGCGATCGCGGCGTTGCTGGTGG
	orf3_ LA_R	GCACCGTCTTGATCACTCCCAGCTCTCTCGCATGCCCAGCGC
	orf3_ RA_F	CGGAGTGATCAAGACGGTGCTGCGGGTCG

	orf3_ RA_R	GACATGATTACGAATTCGCCATCTCGAACACGGCGCTGATGG
orf2+orf3	orf23_ LA_F	GACGGCCAGTGCCAAGCTTGGACGTCGACCTGGTCGTGGTGGCC
	orf23_ LA_R	CCGACTCCACCAGTTCCGTTATTTCAGGTGCTCCTTCGGGTGAGATG
	orf23_ RA_F	CCTGGCGGAACTGGTGGAGTCGGCGGACG
	orf23_ RA_R	GACATGATTACGAATTCGGCGCTGCTCGTTGGCGAGACGGCC
orf4	orf4_ LA_F	GACGGCCAGTGCCAAGCTTGCTGTTCCTCGCGGCGGTGTACTGG
	orf4_ LA_R	CAACCGGGCACGCCCGACGCCGCGTTGTCACGTCGTGCGCAGCG
	orf4_ RA_F	GGATCGTCGGGCGTGCCCGGTTGCTGTGC
	orf4_ RA_R	GACATGATTACGAATTCGCGGGTACACCGTCTGCGCCGTGAC
orf5	orf5_ LA_F	GACGGCCAGTGCCAAGCTTGACGACGCCCGTAACGCCCGGCAG
	orf5_ LA_R	CCGTCCGCCGTGGGAGAAGGGTCGCGGCGCAGGAAACGGAAGG
	orf5_ RA_F	CCTTCTCCCACGGCGGACGGTCGTGGG
	orf5_ RA_R	GACATGATTACGAATTCGCCGTACGGGGCGGATCAGTCGTGC
nucR	nucR_LA_F	GACGGCCAGTGCCAAGCTTCCCATGTCAGACCGAGTAGCCCAG
	nucR_LA_R	CACCACCGCCTCCACCATGCTCTACGGCTCGTCACAGGAATGTC
	nucR_RA_F	CCATGGTGGAGGCGGTGGTGCTGCCG
	nucR_RA_R	GACATGATTACGAATTCGGCGACATCGTTATCCTCCGTCAGG
nucM	nucM_LA_F	GACGGCCAGTGCCAAGCTTCCGTTTCCTGCGCCGCGACCGG
	nucM_LA_R	CTCGTCGCTGTCGGCCTCCCCGGCGGTGAACATTTACCGTCG
	nucM_RA_F	GGAGGCCGACAGCGACGAGCAGCGTCTGG
	nucM_RA_R	GACATGATTACGAATTCGCCAGCACGGTCCGCTCGTCGACCC
nucG	nucG_LA_F	GACGGCCAGTGCCAAGCTTGCGTCAGTCGGCGGGAGTGTGCGC
	nucG_LA_R	CGGATCCGGTTGGTGTGCGGATGCGTCGGGACGGCGGAAAGG
	nucG_RA_F	GCACACCAACCGGATCCGGACGGTCGAC
	nucG_RA_R	GACATGATTACGAATTCGCGGCGAGGACCTGGCGGGCTGG
nucN	nucN_LA_F	GACGGCCAGTGCCAAGCTTCTCCACGGCCGTCATATACGGAC
	nucN_LA_R	CAGGATGCCGGTGAGGCAGGTCAGACCGAGTAGCCCAGCGC
	nucN_RA_F	CTGCCTCACCGGCATCCTGCGCCGCGACG
	nucN_RA_R	GACATGATTACGAATTCGCTGCGGCCGGTACGCAGACGCTC
nucI	nucI_LA_F	GACGGCCAGTGCCAAGCTTGACTCCACGCCGTACAGGATGATG
	nucI_LA_R	GGTCGTTGGGGTACCAGCCCCTTTCGCGGGTTGCTGATGGAGC
	nucI_RA_F	GGCTGGTACCCCAACGACCACTCCGGC
	nucI_RA_R	GACATGATTACGAATTCGCTGATCATCGTCGGTGACGACGTGG
nucJ	nucJ_LA_F	GACGGCCAGTGCCAAGCTTGTCCCAGCGCACCGCCGACAGC
	nucJ_LA_R	GTTCAGGTGGTCGATCTCCGGATCTCTCTAGCTGGACATGCG
	nucJ_RA_F	GGAGATCGACCACCTGAACTGGCTGTCGG
	nucJ_RA_R	GACATGATTACGAATTCGCTCGAAGTACTCCTCGCTGTCG
nucK	nucK_LA_F	GACGGCCAGTGCCAAGCTTGCGGTCCTGCACGTGCTGCGCAAGG
	nucK_LA_R	GGACCTGTGGTGCTCGAGCTTAGGCCTCGTTGACGCCGGCCAGCGAG
	nucK_RA_F	CATGCTCGAGCACCACAGGTCCGCCGACG
	nucK_RA_R	GACATGATTACGAATTCGGCGTTGTCGAGCAGTTCGAGCACC
nucL	nucL_LA_F	GACGGCCAGTGCCAAGCTTCGAGGCCAAGCGCACCCGGCCG
	nucL_LA_R	CTCGCAGCCGGCCTTGGCGTACAGGTCGTGTCGGATGGTGTCG
	nucL_RA_F	GCCAAGGCCGGCTGCGAGCAGACGCGGAC
	nucL_RA_R	GACATGATTACGAATTCGCGACGCGGTCGAGGCGGGTGGAGG
nucQ	nucQ_LA_F	GACGGCCAGTGCCAAGCTTGACAAGTGCAGCGAGCCCGAGGTG
	nucQ_LA_R	GTCGGCCTGCCCCTCCTCGGTTCTCCTCTACTGCGCGGGG
	nucQ_RA_F	GAGGAGGGGCAGGCCGACACCGCGAGCTG
	nucQ_RA_R	GACATGATTACGAATTCGGTGCAACGACGACGTGGGGTCGGG
nucP	nucP_LA_F	GACGGCCAGTGCCAAGCTTGCTGCAAGAGCACGTTCATGGTGG
	nucP_LA_R	CAGGGAGACCACCGCGGTCATTCGTCGGCTCCTCCAGGGATGAGGGC
	nucP_RA_F	GTGATCAACCGGTACGCCGCCGCGCACAAC
	nucP_RA_R	GACATGATTACGAATTCGCTCGAAGCGCAGCGTCAGGCTGTCC
	nucO_LA_F	ACGGCCAGTGCCAAGCTTAACTGCTCGACAACGCGGTGGACGA

nucO	nucO_LA_R	TCAGATCCAGTGTGCGGGGTCGGTTCACAGGGGCTTCCTGAGCGAGT
	nucO_RA_F	ACCGACCCCGCACACTGGATCTGACGGA
	nucO_RA_R	TGACATGATTACGAATTCTACGTACGGTCGATGACCTGGT
nucV	nucV_LA_F	GACGGCCAGTGCCAAGCTTGACCCACCGTGTGCTGGACGCCG
	nucV_LA_R	GAGTTCGATCGCCGCCTCGCCGTCAGATCCAGTGTGCGGGG
	nucV_RA_F	CGAGGCGGCGATCGAACTCGTCCACTCCAC
	nucV_RA_R	GACATGATTACGAATTCGGTCGTCCGCCGACACCGTCGGCTGG
nucPNP	nucPNP_LA_F	GACGGCCAGTGCCAAGCTTGGTACTACGACCTGCCGCCCGACCTG
	nucPNP_LA_R	CAGTACGGGTCGGCCATGGCTGCCTGCCCGGTGCGGAGTTCATCC
	nucPNP_RA_F	CCATGGCCGACCCGTACTGTTCCACCGG
	nucPNP_RA_R	GACATGATTACGAATTCGCCGGACGACCATGTCCACGAAGGCCC
orf9	orf9_ LA_F	CGGCCAGTGCCAAGCTTCAGGAGCCGGACTCCGGTCATG
	orf9_ LA_R	CAGCTGTGGTCGCCCTCGGACCTGAACACCGGCTGCATG
	orf9_ RA_F	GAGGGCGACCACAGCTGGATGGGCATCC
	orf9_ RA_R	GACATGATTACGAATTCGCCGGCTCCGACCAGGTCTCCTTCC

Table S3. List of primers used for knockout verification and plasmid sequencing.

TARGET GENE	PRIMER NAME	PRIMER SEQUENCE
nucA	nucA_vF1	CAAGGTCGAGCCGCTGGAGGAAGGTC
	nucA_vF2	CTACATCGAGCGGGTGCTCAACCGCTTCC
	nucA_vR	GTGTCCTCATCGACGGTCGCGACGGTCG
$nucW_1$ (c1)	nucW1_vF1	CCGAGCTGGACGTGTGGCAGTACATC
	nucW1_vF2	GGTATGTCCTTCACGATCGCCTTGAC
	nucW1_vR	CATGACGACCGTCGCGACCGTCGATG
$nucW_1$ (c2)	nucW2_vF1	GGTGAGGGTCGATCCGTCGGCCGG
	nucW2_vF2	GAGATCCAGTTGAGCAGGGCCACGTC
	nucW2_vR	CCGAAGGACGGCGAACCGGTGGAG
orf(-6)	orf-6_vF1	GGTTGTCGCCTCCCGAACCGACCAGG
	orf-6_vF2	CCCTACTGATGAGTATTTCGTAGGCTG
	orf-6_vR	GATGAGCTCGATCAGGCAGCGGCCGTC
orf(-5)	orf-5_vF1	CCCTACTGATGAGTATTTCGTAGGCTG
	orf-5_vF2	GGTTGTCGCCTCCCGAACCGACCAGG
	orf-5_vR	CGTGGCTGCCGAGGAACACGAACAGTC
orf(-4)	orf-4_vF1	GGTTCACGGTGCGGATGTTCGAGAGG
	orf-4_vF2	GACGATGAGCACTCTTGACGATCAGCAC
	orf-4_vR	GATCATCAGCGCGAAGCCCAGCGAGCTG
orf(-3)	orf-3_vF1	GTCCCGTGAGCGAAGCGGTGCGGTGAC
	orf-3_vF2	CCGGTTACGTCCTCCTGGTCGGTTCGGG
	orf-3_vR	CGGGTGAGGACGACGGTGCCGTATCGG
orf(-2)	orf-2_vF1	GTGACGGCGTTCCGGCAGTACCGCTAC
	orf-2_vF2	GGCAACGCGCTGGGCGCCTATCTGGC
	orf-2_vR	CGAGATCCGGTGCGCAGTACACGTGC
orf(-1)	orf-1_vF1	CGTTCCGTGTCTATCTGGCGCCTCAGC
	orf-1_vF2	CGAGCTCATCCATGACCACCGGTTCAG
	orf-1_vR	GGACTGTGGGGAGTGTCTGTGTGGCG
orf1	orf1_vF1	GCACTCCGCTGACCGCCGTAGCCGCAC
	orf1_vF2	CCTGGTCGTGCCGAGCGAGATCGACG
	orf1_vR	CCAGCGCGTCGTCGAACGTGGCCACG
nucU	nucU_vF1	GACGTCGCGCAGGTGGAGTTGCGGG
	nucU_vF2	GGAGCATCCTCGATGAACGCAGCCCTC
	nucU_vR	CACAGGAACAGGAAGTCGAAGTGGTGC
orf2	orf2_vF1	GAGCGCAACAGCACGGCCGTGAACCTC
	orf2_vF2	GTGCTCATCACCGTGATCGGTCTGGTG
	orf2_vR	GCTGGTACTTGATGTCCGAGGTGATGC
orf3	orf3_vF1	GACGACTACCGGTACACGTTCTTCGTC
	orf3_vF2	GAGCGCAACAGCACGGCCGTGAACCTC
	orf3_vR	GGCTGATTGGCCCATCACCTTCGTCTAC
orf4	orf4_vF1	GCAGACACACCGTTGACGCCGCCGGTCGG
	orf4_vF2	CTGGACGACTACCGGTACACGTTCTTCG
	orf4_vR	GTCGATGCGGTGCAGCAGGTCGAGCATCG
orf5	orf5_vF1	GGTGCTGTTCCAGTCGTCGTCCGACGTG
	orf5_vF2	CTTCCTGCGGATCGCGGTGGACCTGCTC
	orf5_vR	CCCTCGCGAGTTTCGGTCCGCTCCTGG
nucR	nucR_vF1	GCCTCCACCGGCGGTGAACATTTACC
	nucR_vF2	CATGGTCCTCACCTCGGATGCGTC
	nucR_vR	CTCCGAGCTGGTAGGTGACGGCCAG
nucM	nucM_vF1	GGACTCCGACGAATTCTCTACGGCTCGTC
	nucM_vF2	CAGGAGCGGACCGAAACTCGCGAGG

	nucM_vR	GGAGATCAGTACGACGTGATCGTAAGGGG
nucG	nucG_vF1	GGCATAGGTGGCGAACGAGGCCAG
	nucG_vF2	GGCATAGGTGGCGAACGAGGCCAG
	nucG_vR	GTTCTGGAACCACTCGCTGTACTGCC
nucN	nucN_vF1	GTCGCCTACCAGCAGCGCCAGCTCTC
	nucN_vF2	CGGTGACACCGTCGAGAGCTACGACC
	nucN_vR	GTACGGCTTCCACAACCTGGCCTTCGG
nucI	nucI_vF1	CCTCCAGCAGCAGCTGCACGTGGTC
	nucI_vF2	GCCACGGCGGGAGGAATGTGACTGTCTG
	nucI_vR	CCGGGTAGTAGGTCTCGCTGTAGGC
nucJ	nucJ_vF1	CGGTCCTGCACGTGCTGCGCAAGGAC
	nucJ_vF2	CGCTGCACTTGTCGAGGGTGAAGCGC
	nucJ_vR	GACCACGTGCAGCTGCTGCTGGAGGCC
nucK	nucK_vF1	CTGCACGAGGACGAGTTCCTCAACGTCAGC
	nucK_vF2	GCTGCAAGATGGACTGCTCGTTCTGCTAC
	nucK_vR	CGTTGTACGTGTGCTGTCAGGCGGTGCTGC
nucL	nucL_vF1	CCTACGTACGGTTCCTCCGGCTGAG
	nucL_vF2	GAGCACATGCAGTACTTCCACCCGCAG
	nucL_vR	CGTTGTCCGACCAGACGTCGACGTCC
nucQ	nucQ_vF1	GAGCAGACGCGGACCCGCTCGTAC
	nucQ_vF2	CTCTGCGACTTCCTCGGCGAGGAACTG
	nucQ_vR	GGATGAGGGCGGCATCGTGCTGTTCG
nucP	nucP_vF1	CTGTTCGGCGAACTCGCCGACAGCCTCG
	nucP_vF2	GGTGAACCTCTGCGCCTACGACGAAC
	nucP_vR	CTGCTGCGCCAGCAGGTCGTCGCTGTTG
nucO	nucO_vF1	CGCTGGTGCGTCTACTCGCTCAGGAAG
	nucO_vF2	CCAGATGTTCCGTGAGGAGGAGTTCCG
	nucO_vR	CTCAGATCCAGTGTGCGGGGTCGGTG
nucV	nucV_vF1	CCTGACGCTGCGCTTCGAGGACCTC
	nucV_vF2	CGCTGGTGCGTCTACTCGCTCAGGAAG
	nucV_vR	GCCTGGACGCAGTCGCTCTTCACGAG
nucPNP	nucPNP_vF1	CGTCCGCACACACCACATGACCGGAGAC
	nucPNP_vF2	GTTCGCCGACACCGTCGACATCCTGTCC
	nucPNP_vR	CACGGCGAGGACCTCCTCGTGCGTGACGC
orf9	orf9_vF1	CCTCAGTTGAACGCTCCCCCGGTTG
	orf9_vF2	GGATCTCGTTCACGACGCGCTCCTCAC
	orf_vR	GTAGACGACGAACTGCAGGAACCGCTC

Table S4. List of primers used to construct complementation plasmids. Uninducible (“native”) plasmids where *tipA* promoter is replaced with a native one are denoted with (noInd), his and NH refer to the presence and absence of a His6-tag on the resulting gene product, respectively.

TARGET GENE(S)	PRIMER NAME	PRIMER SEQUENCE
nucA	nucA_F	GAAGGGAGCGGACCATGGCAGCCTCATCGACGAACCAGGAGAC
	nucA_R	GTCGACGGAGCTCGAATTCTCACAGAAGTCCCCTTTCGCTGAG
	nucAnoInd_F	CAGATGGAGTTCTGAGGTCGACGAGTCCGTGACCAGGGCCGAC
orf(-3)	orf-3_F	GAAGGGAGCGGACCATGGTTGTGCAAGCCGTTTCAGAACGTGTTC
	orf-3_R	GCTCGAGTGCGGCCGCAAGGGCGGTCCGGCCATCGCCCGGCGTG
	orf-3noInd_F	CAGATGGAGTTCTGAGGTCGGCACACGGTGTCCGCAAGCAGGG
	orf-3NH_R	GTCGACGGAGCTCGAATTCTCAGGCGGTCCGGCCATCGCCCGG
orf2	orf2_F	GAAGGGAGCGGACCATGGTCGATCCGAGGATGCCCGAGAGCG
	orf2his_R	GCTCGAGTGCGGCCGCAAGGCTCTCTCGCATGCCCAGCGCGGTG
	orf2noInd_F	CAGATGGAGTTCTGAGGTCCATCGTCCTCGCGTCGGTCACCTAC
	orf2NH_R	GTCGACGGAGCTCGAATTCTCAGCTCTCTCGCATGCCCAGCGC
orf3	orf3_F	GAAGGGAGCGGACCATGGCCGAGTCCCCTCCCCCGCGCG
	orf3his_R	GCTCGAGTGCGGCCGCAAGCGTCGTGCGCAGCGCGGGCGGGAG
	orf3noIndF	CAGATGGAGTTCTGAGGTCCAGCATCGACCGGGCGTTCGTCAG
	orf3NH_R	GTCGACGGAGCTCGAATTCTCACGTCGTGCGCAGCGCGGGCGG
nucU+orf2	nucUnoInd_F	CAGATGGAGTTCTGAGGTCGCGTCCTGGAGGTCCGGGTCGACG
orf2+orf3	orf2noInd_F	CAGATGGAGTTCTGAGGTCCATCGTCCTCGCGTCGGTCACCTAC
	orf3NH_R	GTCGACGGAGCTCGAATTCTCACGTCGTGCGCAGCGCGGGCGG
	orf3orf2_F	CTGAGATCCGGCTGCTAACGAAGGGAGCGGACCATGGTCGATCC
	orf3orf2_R	CTAACGTCTGGAAAGACGACTCAGTGGTGGTGGTGGTGGTGCTCG
nucN	nucN_F	CCGCGCGGCAGCCATATGGGCCGGTCCGACACGACACCC
	nucN_R	GTCGACGGAGCTCGAATTCGTTGCGGCGTCACACGTCACATCG
nucK	nucK_F	GAAGGGAGCGGACCATGGCAGGCCGACTCGGTCCCGTTCTC
	nucK_R	GTCGACGGAGCTCGAATTCGTTGTACGTGTGCTGTCAGGCGGTG
nucQ	nucQ_F	GAAGGGAGCGGACCATGGCGCACATCAGTGACCGTCTCGC
nucO	nucQ_R	GCTCGAGTGCGGCCGCAAGCTCATCGGCTCCTCCAGGGATGAG
	nucO_F	GAAGGGAGCGGACCATGGCCCGCACCGGCCACCCGCATCCC
	nucO_R	GCTCGAGTGCGGCCGCAAGGATCCAGTGTGCGGGGTCGGTGTAG

Table S5. List of ORFs involved in the biosynthesis of nucleocidin in *S. calvus* and *S. virens*.

ORF	**Annotation**	**Accession number in *S. calvus***	**Accession number in *S. virens***	**Protein sequence similarity**
orf-3	Cysteine dioxygenase	WP_233452298	MYS29810	100.00%
orf-2	NUDIX hydrolase	WP_142191275	MYS29809	98.56%
orf-1	Protein of unknown function	WP_142191276	MYS29808	98.7%
orf1	Oxidoreductase	WP_142191277	MYS29807	99.70%
nucU	Cation: proton antiporter	WP_233452300	MYS29806	99.34%
orf2	Hypothetical protein	WP_142191278	MYS29805	100.00%
orf3	Histidine phosphatase family protein	WP_233452302	MYS29804	98.92%
orf4	Transcriptional regulatory protein	WP_267905327	MYS29803	98.44%
orf5	Aminoglycoside phosphotransferase	WP_182674813		
nucR	Inner membrane metabolite transport protein YhjE	WP_182674811	MYS30223	99.77%
nucM	Hypothetical protein	WP_142191281		
nucG	Sulfatase	WP_142191282		
nucN	Amidinotransferase	WP_142191283	MYS28573	99.39%
nucI	Sulfatase	WP_142191284	MYS28574	100.00%
orf6	StrR-like transcriptional regulator	WP_142191285	MYS28575	99.15%
nucJ	Radical SAM superfamily protein	WP_142191286	MYS28576	100.00%
nucK	Sulfotransferase domain protein	WP_142191287	MYS28577	100.00%
nucL	SAM-dependent methyltransferase	WP_202432158	MYS29784	99.60%
nucQ	Rubrerythrin	WP_142191288	MYS29783	100.00%
nucP	Methyltransferase	WP_142191289	MYS29782	99.85%
nucO	Sulfotransferase	WP_233452305	MYS29781	100.00%
nucV	Adenine phosphoribosyl-transferase	WP_142191290	MYS29780	100.00%
orf7	LuxR family transcriptional regulator	WP_142230912	MYS29779	99.44%
nucPNP	S-methyl-5'-thioadenosine phosphorylase	WP_142191292	MYS29778	100.00%
orf8	Lycopene cyclase	WP_182674809	MYS28276	99.56%
nucGT	Glucosyltransferase	WP_142191293	MYS28275	100.00%
orf9	Hypothetical protein	WP_142191294	MYS28274	99.84%
orf10	Protein kinase domain containing protein	WP_142191295	MYS28273	99.72%
nucBGS	Beta-glucosidase	WP_142191296	MYS28272	100.00%

3. Supplementary figures

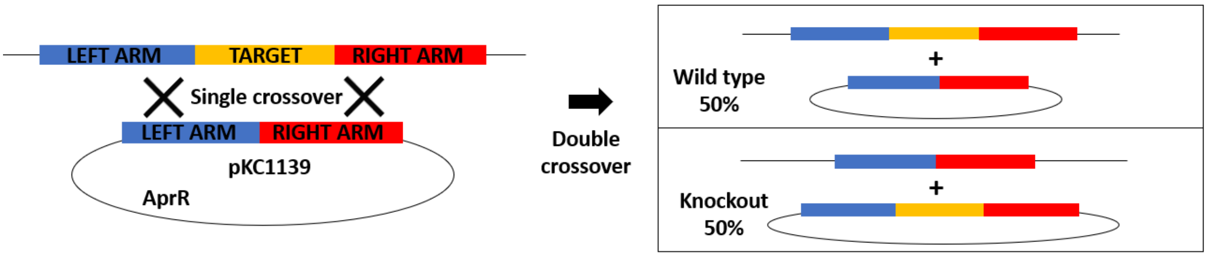

Figure S1. Gene deletion strategy used in this study. "Target" region denotes ~2/3 of gene to be deleted, including the start codon, so that the "right arm" region contains the terminal portion (usually about a third) of the gene. Single crossover occurs when the cells containing the knockout plasmid are grown in the presence of apramycin at temperature >32°C. After several generations on plates without the antibiotic and at <32°C colonies are screened for apramycin resistance – the genotype of the sensitive colonies is verified with PCR as the expected ratio of knockout to wild type colonies is 1:1.

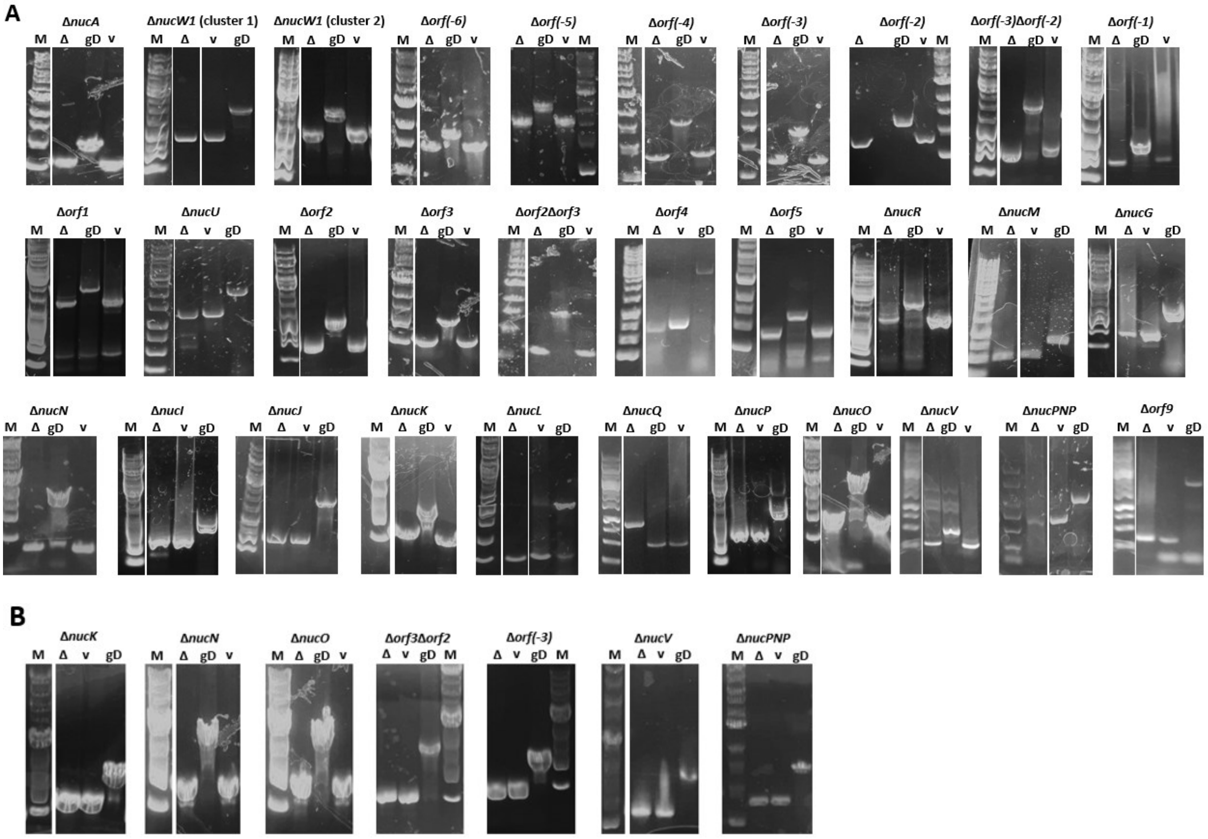

Figure S2. PCR verifications of individual gene knockouts. A – knockouts in *S. calvus*, B – in *S. virens*. Genomic DNA (gD) and the vector used in generating the knockout (v) were used as templates in control reactions.

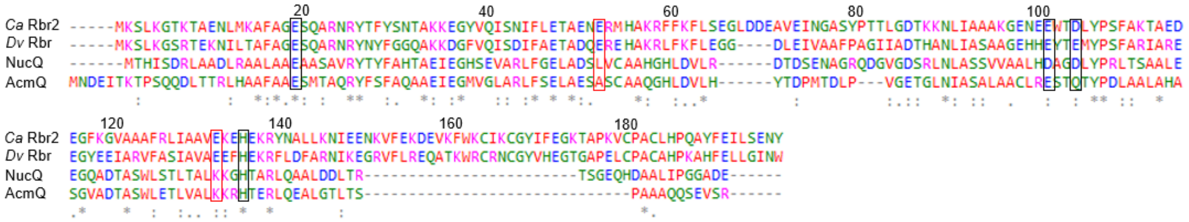

Figure S3. Sequence alignment of NucQ against AcmQ and two representative rubrerythrin proteins – Rbr2 from *Clostridium acetobutylicum* and Rbr from *Desulfovibrio vulgaris*. Conserved metal-binding sites are highlighted, those missing in NucQ and AcmQ are shown in red.

LC-MS data of the standard 9~11 can be found in ref [3]

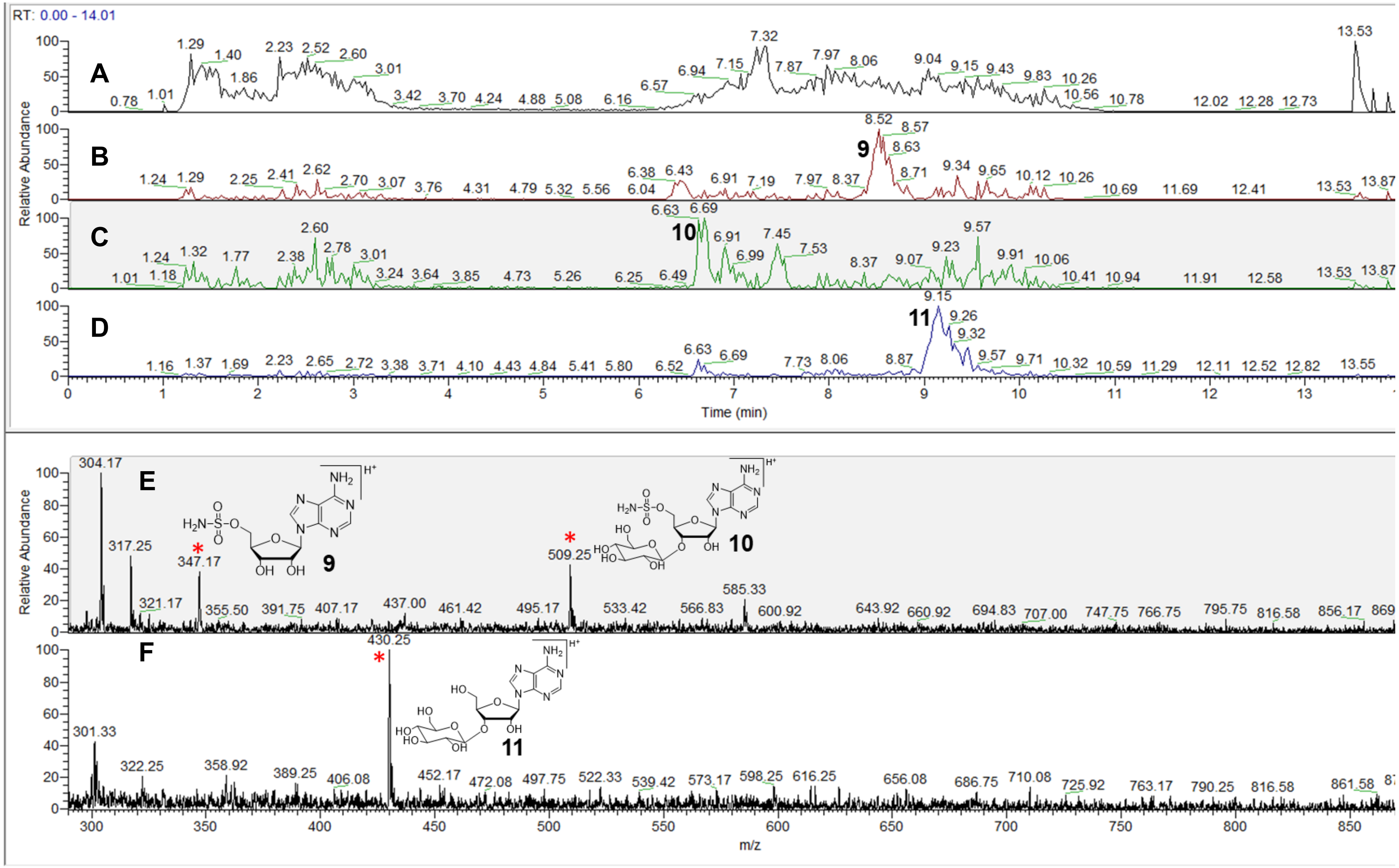

Figure S4. LC-MS analysis of: (A) total ion of *S. calvus Δorf2Δorf3* media extract; (B-D) ion extraction of **9**, **10** and **11**; (E) molecular ions of **9** and **10**; (F) molecular ions of **11**.

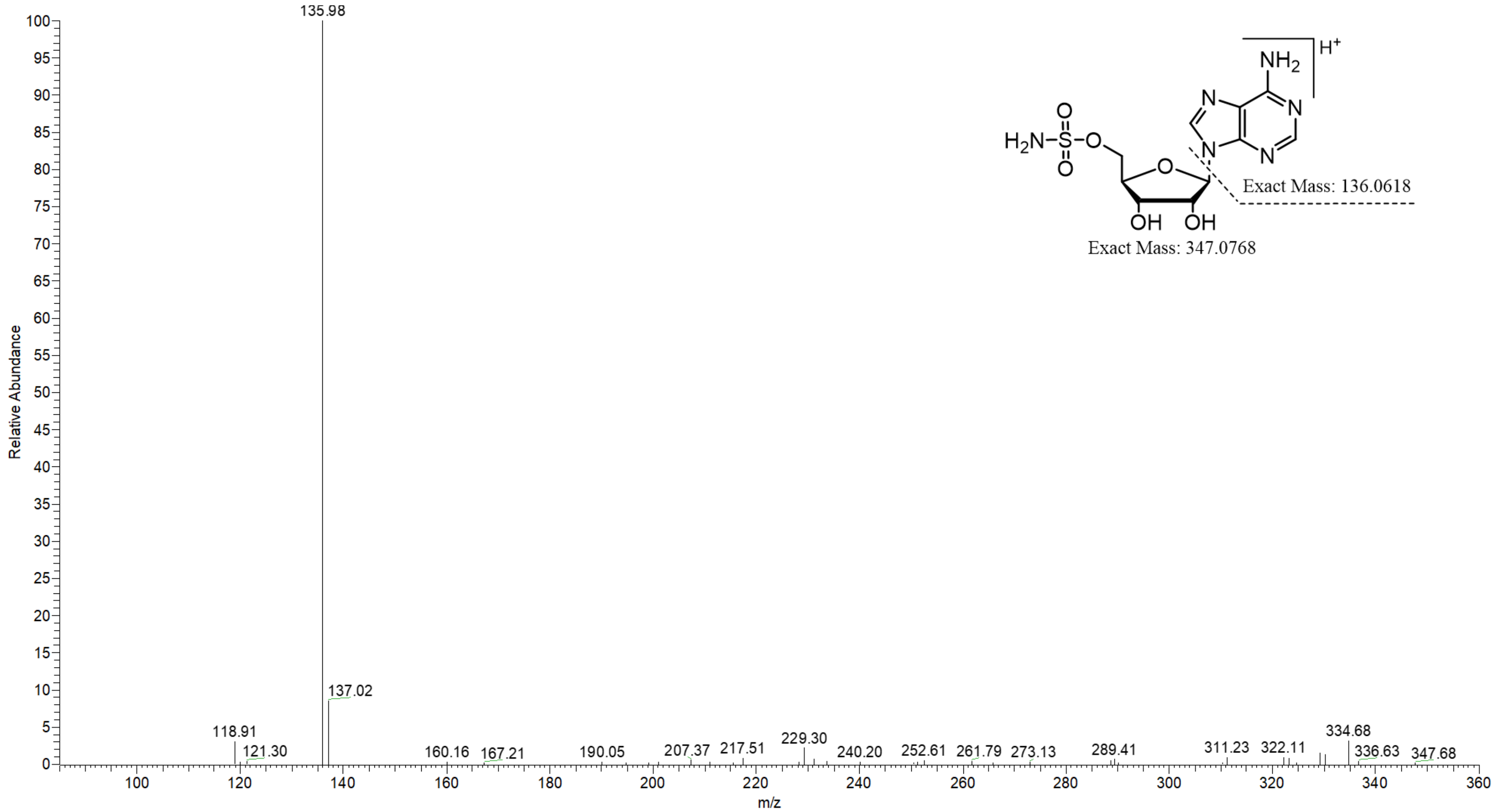

Figure S5. Daughter ion $[M+H]^+$=135.98 amu of **9** extracted from total ions of *S. calvus ΔorfΔorf3* media extract.

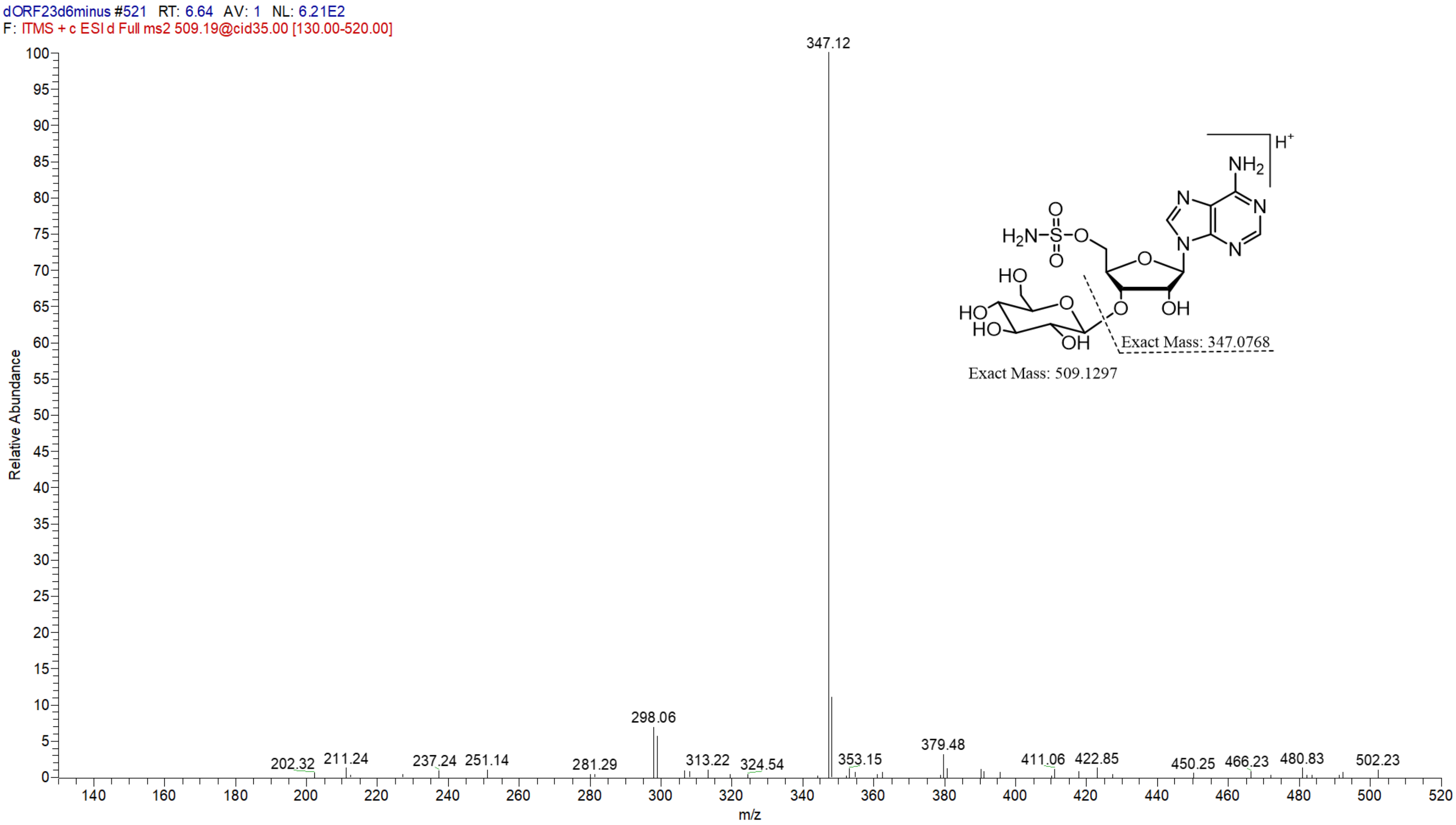

Figure S6. Daughter ion $[M+H]^+$=347.12 amu of **10** extracted from total ions of *S. calvus Δorf2Δorf3* media extract.

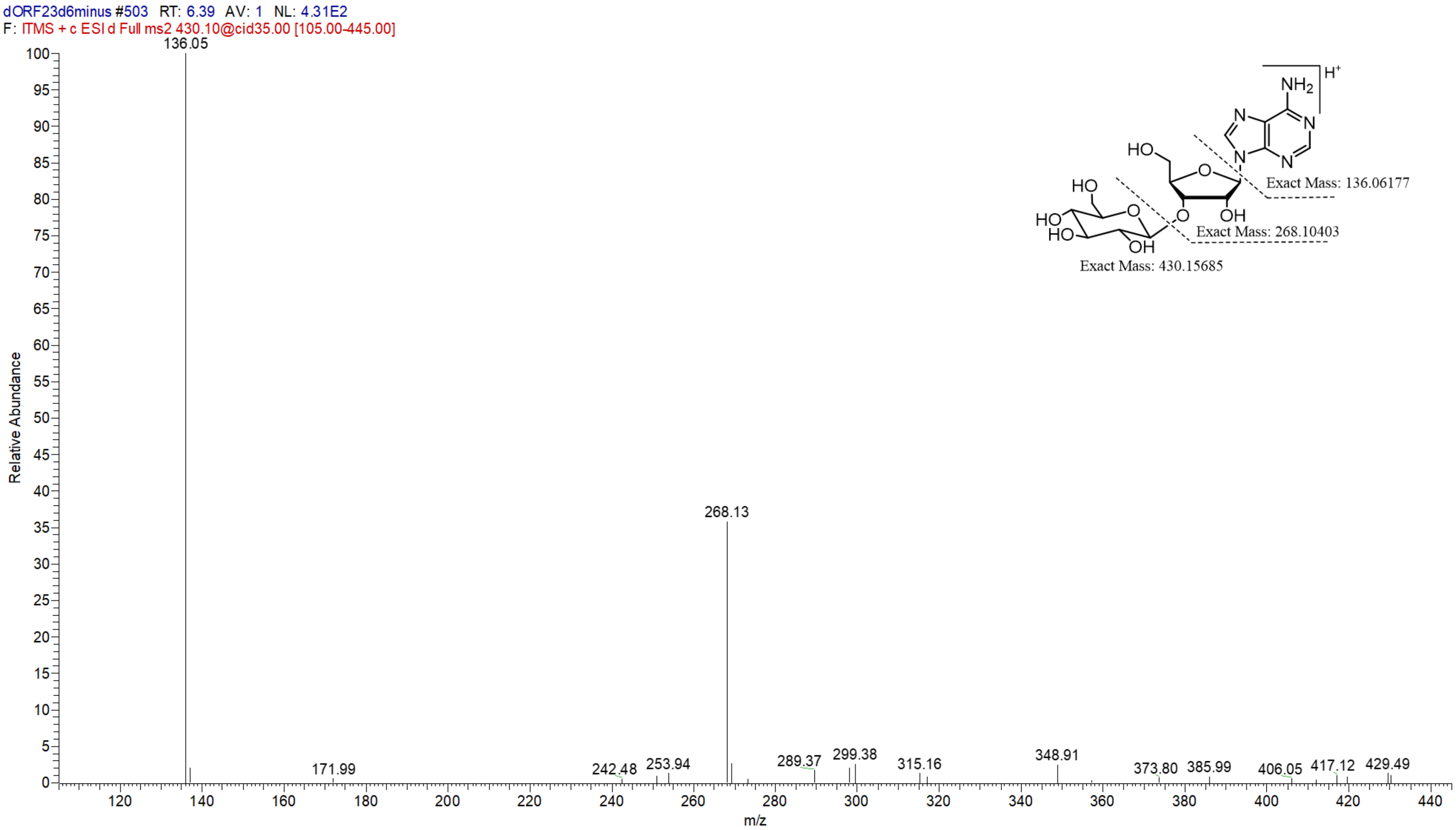

Figure S7. Daughter ions $[M+H]^+$=136.05 and $[M+H]^+$=268.13 amu of **11** extracted from total ions of *S. calvus Δorf2Δorf3* media extract.

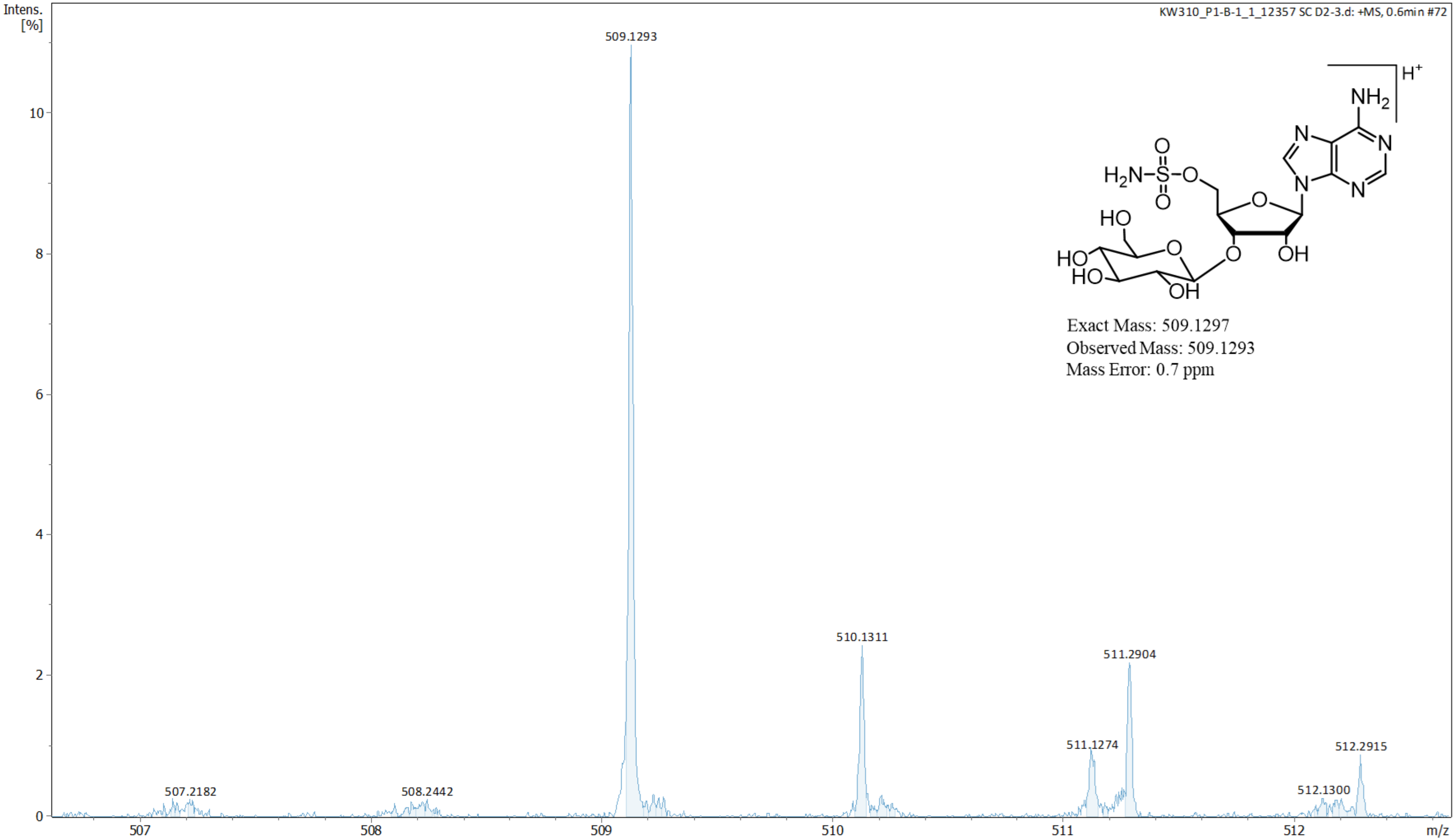

Figure S8. High resolution MS of **10** extracted from total ions of *S. calvus Δorf2Δorf3* media extract.

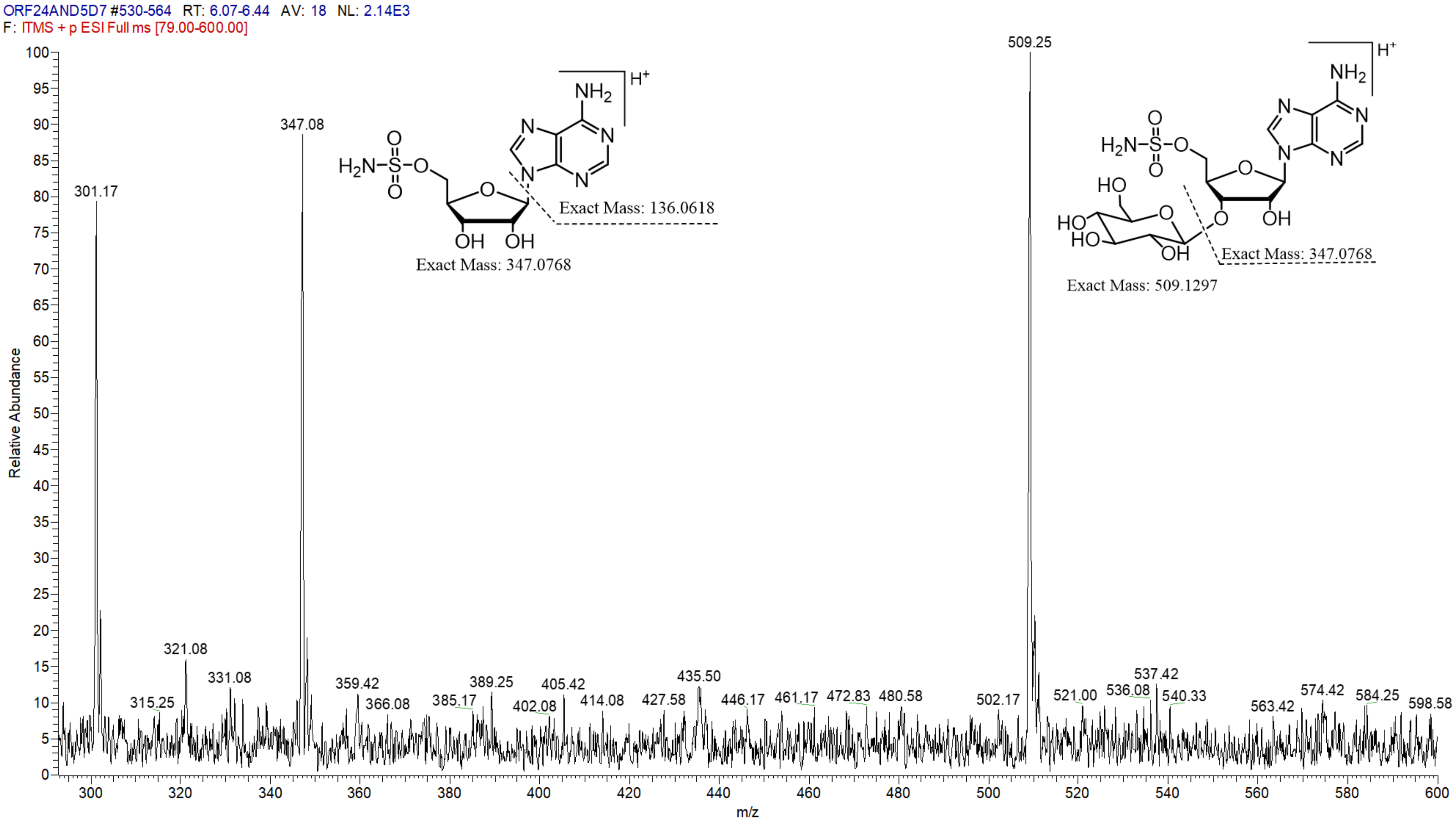

Figure S9. Extracted molecular ions of **9** and **10** from *S. calvus* Δ*orf2* media extract.

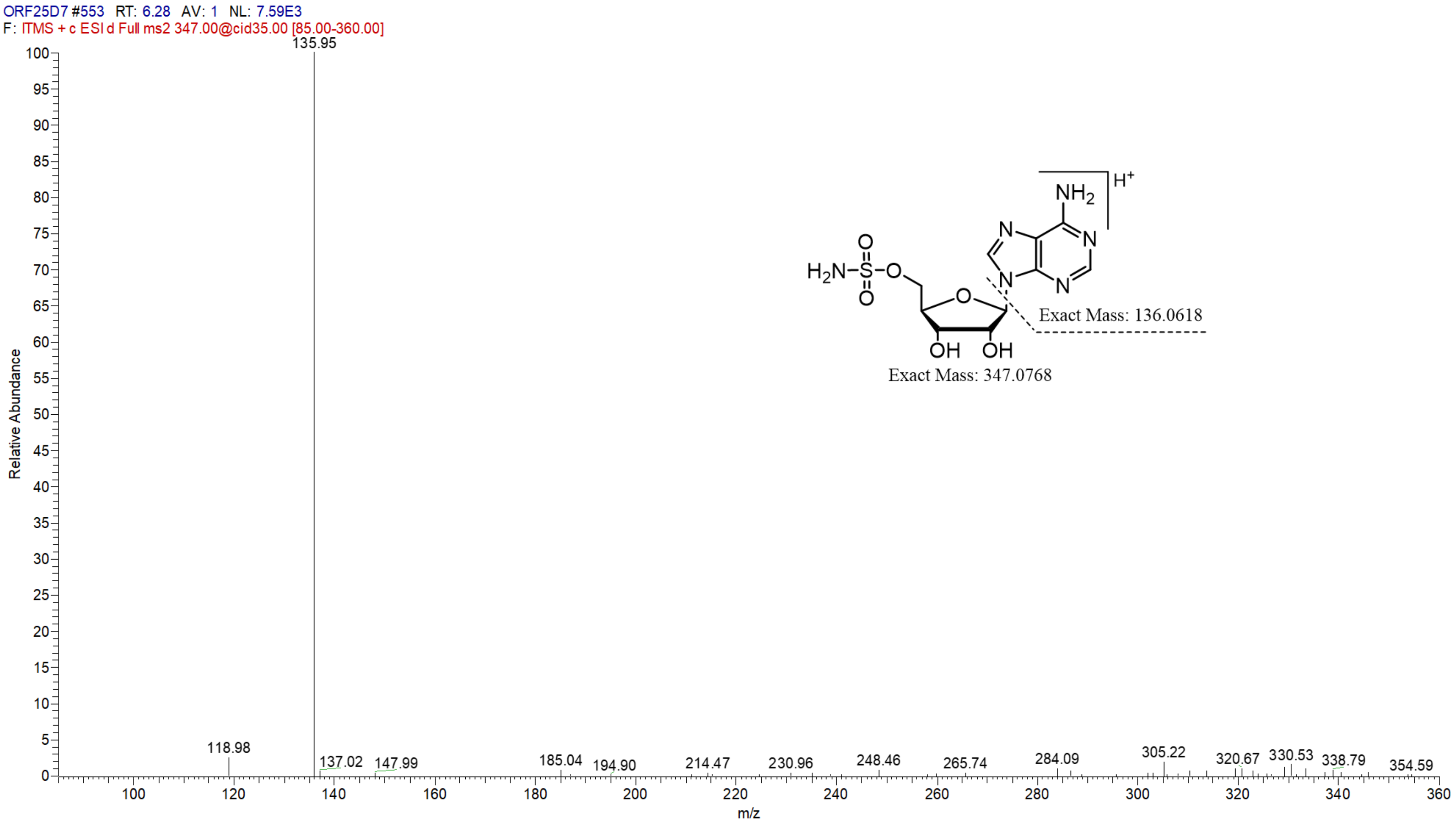

Figure S10. Daughter ion $[M+H]^+$=135.98 amu of **9** extracted from total ions of *S. calvus* Δ*orf2* media extract.

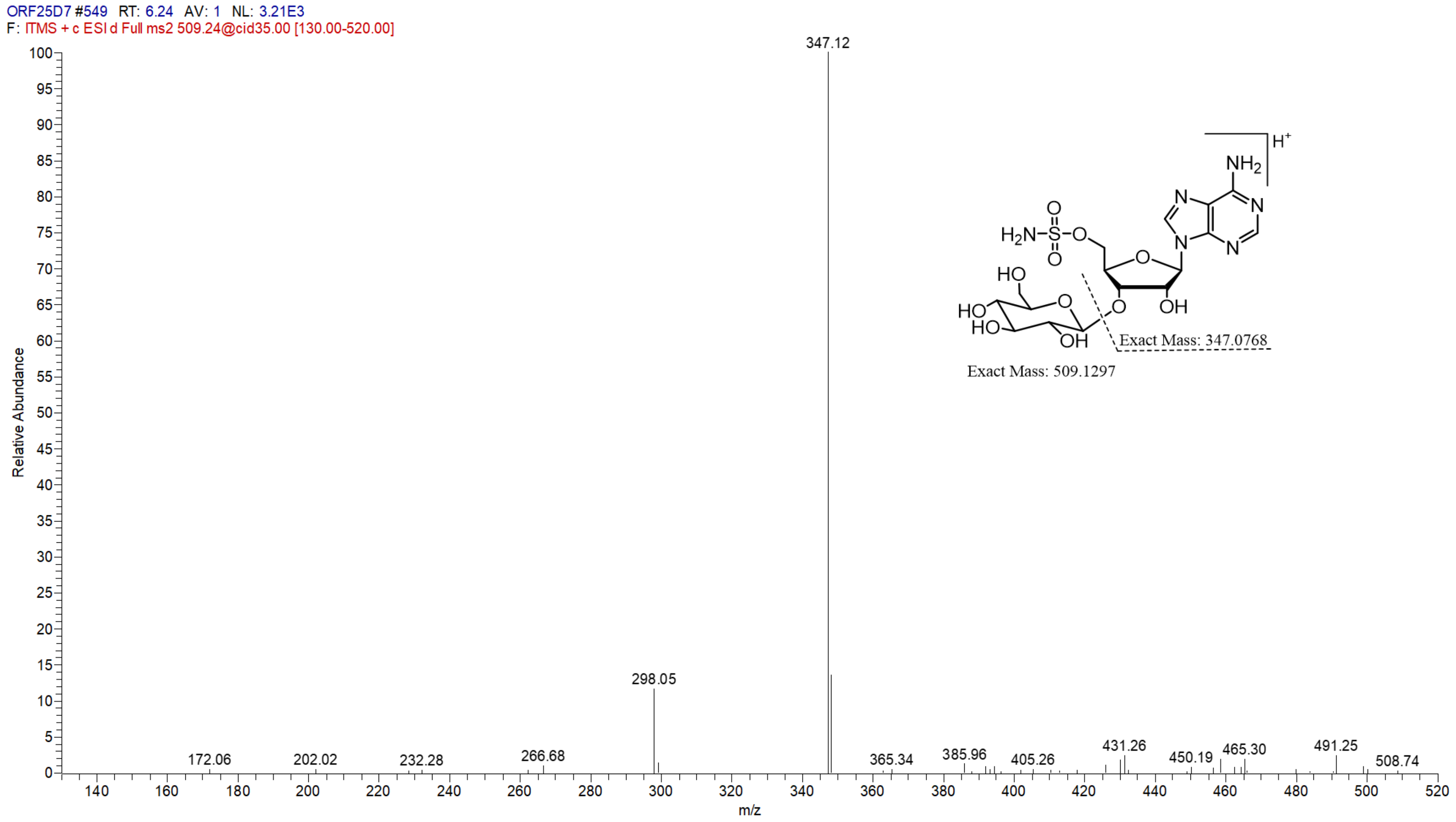

Figure S11. Daughter ion $[M+H]^+$=347.12 amu of **10** extracted from total ions of *S. calvus* Δ*orf2* media extract.

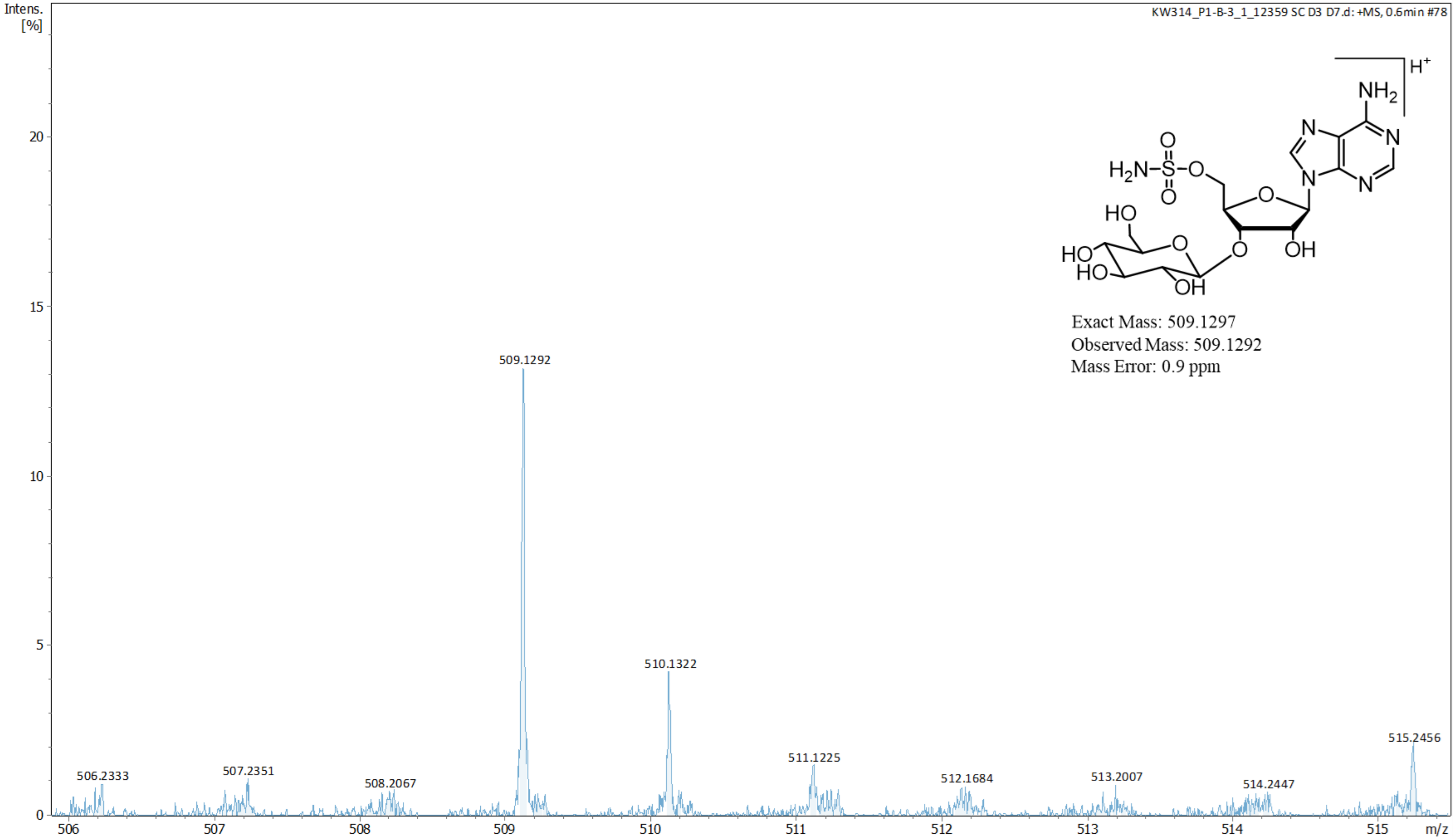

Figure S12. High resolution MS $[M+H]^+$=509.1292 amu (error=0.9 ppm) of **10** from *S. calvus* Δ*orf(-3)* media extract.

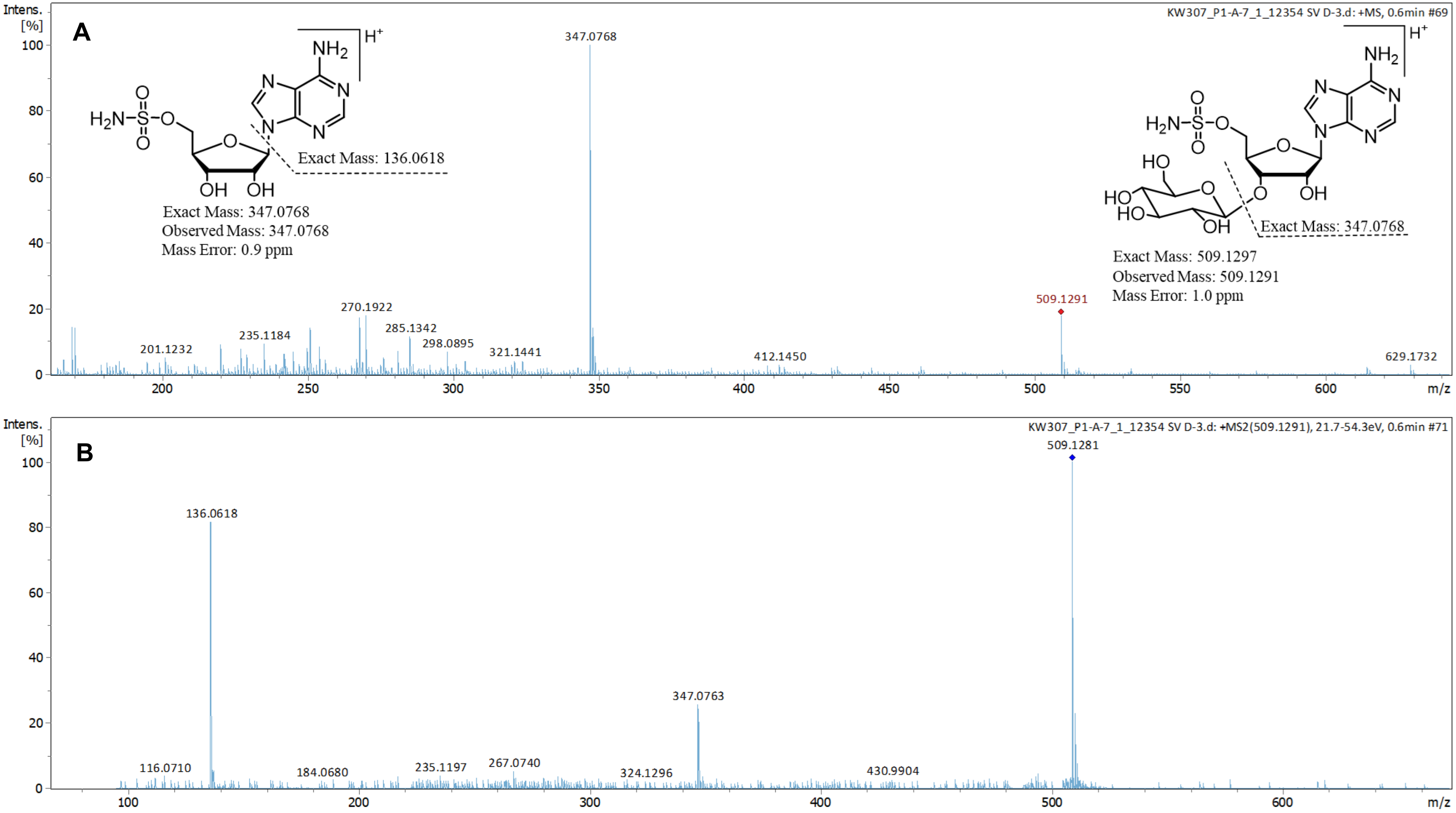

Figure S13. High resolution MS (A) and secondary MS (B) of **10** from *S. virens* Δ*orf(-3)* media extract.

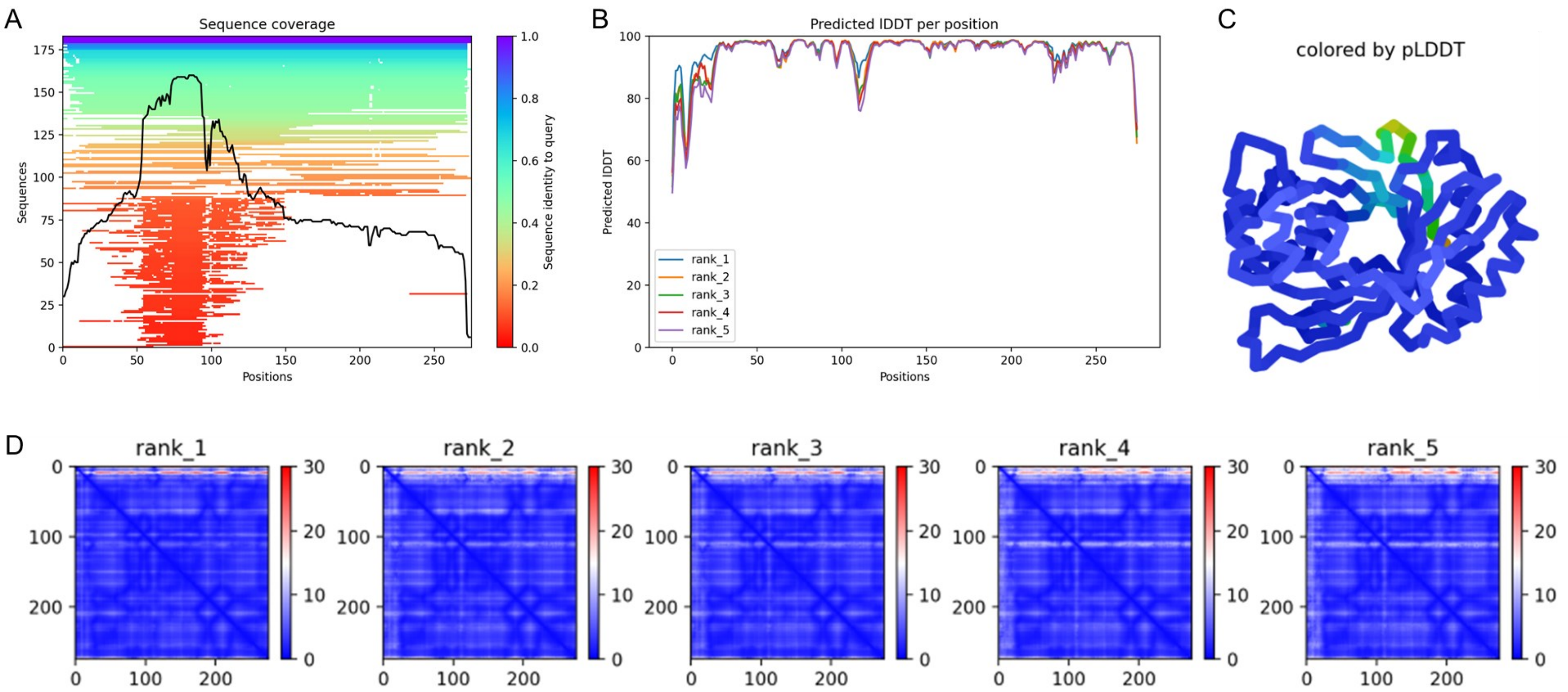

Figure S14. Assessment of AlphaFold2-generated structure of Orf2. A – sequence coverage plot, B – predicted local distance difference test plot (pLDDT), C – Orf2 structure coloured according to pLDDT, D – predicted aligned error plot (PAE).

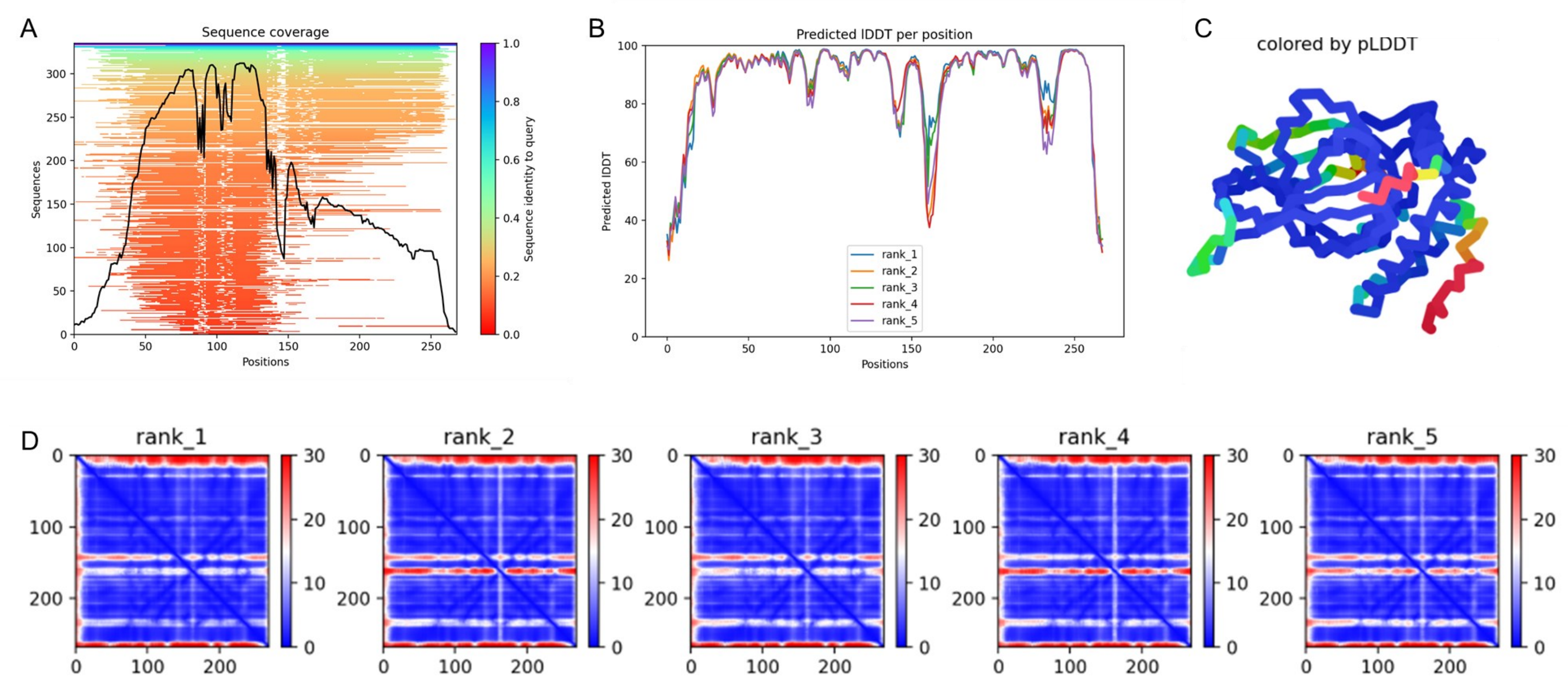

Figure S15. Assessment of AlphaFold2-generated structure of Orf(-3). A – sequence coverage plot, B – predicted local distance difference test plot (pLDDT), C – Orf(-3) structure coloured according to pLDDT, D – predicted aligned error plot (PAE).

5. References

[1] M. Bierman, R. Logan, K. O'Brien, E.T. Seno, R. N. Rao and B. E. Schoner, *Gene*, **1992**, *116*, 43–49

[2] G. Muth, *Appl Microbiol Biotechnol.*, **2018**, *102*, 9067-9080

[3] X. Feng, D.Bello and D. O'Hagan, *RSC Adv.*, **2021**, *11*, 5291-5294
